# Approval-based shortlisting

**DOI:** 10.1007/s00355-023-01482-2

**Published:** 2023-08-11

**Authors:** Martin Lackner, Jan Maly

**Affiliations:** DBAI, TU Wien, Vienna, Austria

## Abstract

Shortlisting is the task of reducing a long list of alternatives to a (smaller) set of best or most suitable alternatives. Shortlisting is often used in the nomination process of awards or in recommender systems to display featured objects. In this paper, we analyze shortlisting methods that are based on approval data, a common type of preferences. Furthermore, we assume that the size of the shortlist, i.e., the number of best or most suitable alternatives, is not fixed but determined by the shortlisting method. We axiomatically analyze established and new shortlisting methods and complement this analysis with an experimental evaluation based on synthetic and real-world data. Our results lead to recommendations which shortlisting methods to use, depending on the desired properties.

**Supplementary Information:**

The online version contains supplementary material available at 10.1007/s00355-023-01482-2.

## Introduction

Shortlisting is a task that arises in many different scenarios and applications: given a large set of alternatives, identify a smaller subset that consists of the best or most suitable alternatives. Prototypical examples of shortlisting are awards where a winner must be selected among a vast number of eligible candidates. In these cases, we often find a two-stage process. In a first shortlisting step, the large number of contestants (books, films, individuals, etc.) is reduced to a smaller number. In a second step, the remaining contestants can be evaluated more closely and one contestant in the smaller set is chosen to receive the award.

Both steps may involve a form of group decision making (voting), but can also consist of a one-person or even automatic decision. For example, the shortlist of the Booker Prize is selected by a small jury (The Man Booker Prize 2018), whereas the shortlists of the Hugo Awards are compiled based on thousands of ballots (The Hugo Awards 2019). Similarly, the Baseball Writers’ Association of America selects the new entries into the Baseball Hall of Fame by voting. In that case, any candidate with at least 75% approval enters the hall of fame, without a second round. Another very common application of shortlisting is the selection of most the promising applicants for a position who will be invited for an interview (Bovens 2016; Singh et al. 2010). Apart from these prototypical examples, shortlisting is also useful in many less obvious applications like the aggregation of expert opinions for example in the medical domain (Gangl et al. 2019) or in risk management and assessment (Tweeddale et al. 1992). Shortlisting can even be used in scenarios without agents in the traditional sense, for example if we consider features as voters to perform an initial screening of objects, i.e., a feature approves all objects that exhibit this feature (Faliszewski et al. 2020).

In this paper, we consider shortlisting as a form of collective decision making. We assume that a group of voters announce their preferences by specifying which alternatives they individually view worthy of being shortlisted, i.e., they file approval ballots. In practice, approval ballots are commonly used for shortlisting, because the high number of alternatives that necessitates shortlisting in the first place precludes the use of ranked ballots.^1^ Furthermore, we assume that the number of alternatives to be shortlisted is not fixed (but there might be a preferred number), as there are very few shortlisting scenarios where there is a strong justification for an exact size of the shortlist. Due to this assumption, we are not in the classical setting of multi-winner voting (Kilgour and Marshall 2012; Faliszewski et al. 2017; Lackner and Skowron 2023), where a fixed-size committee is selected, but in the more general setting of multi-winner voting with a variable number of winners (Kilgour 2010, 2016; Faliszewski et al. 2020).

Moreover, in this paper, we focus on the case where the goal of the shortlisting process is determining the *objectively* best or most deserving candidates. This means that we implicitly assume that all candidates have a true value or quality that is the same for all voter. However, voters have only a noisy perception of these values. In this sense, our model is similar to the epistemic interpretation of voting rules as maximum likelihood estimators (Elkind and Slinko 2016) or to common-value auctions (Klemperer 1999; Shoham and Leyton-Brown 2009), where the item for sale has the same objective value to all bidders, but the bidders have different beliefs about this value.

In real-world shortlisting tasks, there are two prevalent methods in use: *Multi-winner Approval Voting* (selecting the *k* alternatives with the highest approval score) and threshold rules (selecting all alternatives approved by more than a fixed percentage of voters). Further shortlisting methods have been proposed in the literature (Brams and Kilgour 2012; Kilgour 2016; Faliszewski et al. 2020). Despite the prevalence of shortlisting applications, there does not exist work on systematically choosing a suitable shortlisting method. Such a recommendation would have to consider both expected (average-case) behavior and guaranteed axiomatic properties, and neither have been studied previously specifically for shortlisting applications (cf. Sect. 1.1). Our goal is to answer this need and provide principled recommendations for shortlisting rules, depending on the properties that are desirable in the specific shortlisting process.

In more detail, the contributions of this paper are the following:

- We define shortlisting as a voting scenario and specify minimal requirements for shortlisting methods (Sect. 2). Furthermore, we introduce five new shortlisting methods: *First* $k$-*Gap*, *Largest Gap*, *Top*-$s$-*First*-$k$-*Gap*, *Max-Score*-$f$-*Threshold*, and *Size Priority* (Sect. 3).
- We conduct an axiomatic analysis of shortlisting methods and by that identify essential differences between them. Furthermore, we axiomatically characterize *Approval Voting*, $f$-*Threshold*, and the new *First* $k$-*Gap* rule (Sect. 4).
- We present a connection between shortlisting and clustering algorithms, as used in machine learning. We show that *First* $k$-*Gap* and *Largest Gap* can be viewed as instantiations of linkage-based clustering algorithms (Sect. 6.1).
- In numerical simulations using synthetic data, we approach two essential difficulties of shortlisting processes: we analyze the effect of voters with *imperfect (noisy) perception* of the alternatives and the effect of *biased voters*. These simulations complement our axiomatic analysis by highlighting further properties of shortlisting methods and provide additional data points for recommending shortlisting methods (Sect. 5).
- In addition to synthetic data, we collected voting data from the Hugo Awards, which are annual awards for works in science-fiction. This data set is a real-world application of shortlisting and offers a challenging test-bed for shortlisting rules. Using this data set, we investigate the ability of different shortlisting rules to produce short shortlists without excluding the alternative that actually won the award (Sect. 5).
- An open-source implementation (Lackner and Maly 2022) of all considered shortlisting rules and the numerical experiments is available, including the Hugo data set.
- The recommendations based on our findings are summarized in Sect. 6. In brief, our analysis leads to a recommendation of *Top*-$s$-*First*-$k$-*Gap*, $f$-*Threshold*, and *Size Priority*, depending on the general shortlisting goal and desired behavior.

### Related work

There are two recent papers that are particularly relevant for our work. Both Faliszewski et al. (2020) and Freeman et al. (2020) investigate multi-winner voting with a variable number of winners. In contrast to our paper, the main focus of Faliszewski et al. (2020) lies on computational complexity, which is less of a concern for our shortlisting setting (as discussed later). The paper also contains numerical simulations related to the number of winners (which is one of the metrics we consider in our paper). In the few cases where shortlisting rules are considered,^2^ their results regarding the average size of winner sets agree with our simulations (Sect. 5).

Freeman et al. (2020) study proportionality in multi-winner voting with a variable number of winners. We note that proportional representation of voters is incompatible with our desiderata of shortlisting rules (i.e., proportionality is incompatible with the Efficiency axiom, which we require for shortlisting rules). Thus, the rules and properties considered by Freeman et al. (2020) do not intersect with ours and are difficult to compare with. A simplified separation between our work and theirs is the underlying assumption of fairness: we require that the most deserving candidates are included in the shortlist (fairness towards candidates), whereas proportionality is concerned with fairness towards voters.

Shortlisting can also used to find a compromise between two opposing parties, for example when appointing an arbitrator in a dispute. In this setting, the first party proposes a shortlist. Then, the second party picks their preferred candidate from this list. de Clippel et al. (2014) and Barberà and Coelho (2017) study the optimal length for a shortlist in this setting. Núñez and Laslier (2015) and Barberà and Coelho (2022) propose more complex methods to find a compromise using shortlisting and study these methods as two-player games. This use of shortlisting differs from our setting in that the two parties are antagonistic and aim to maximize their private utilities, while we assume that the decision makers are interested in identifying the (objectively) best alternative.

Finally, shortlisting is also the first step in a Participatory Budgeting process. Rey et al. (2021) studied shortlisting and the specific desiderata that arise in the context of a Participatory Budgeting process in their end-to-end model.

There are two other notable voting frameworks with a variable number of winners. First, shortlisting rules can be viewed as a particular type of *social dichotomy functions* (Duddy et al. 2014; Brandl and Peters 2019), i.e., voting rules which partition alternatives into two groups. Moreover, multiwinner voting with a variable number of winners can be seen as a special case of (binary) *Judgment Aggregation* (List 2012; Endriss 2016) without consistency constraints. However, both of these frameworks treat the set of selected winners and its complement as symmetric. This is in contrast to shortlisting, where we usually expect the winner set to be only a small minority of all available candidates. For this reason, social dichotomy functions and Judgment Aggregation rules are generally not well suited for shortlisting.

It is worth mentioning that shortlisting is is not only studied as a form of collective decision making but also studied as a model of individual decision making. Manzini and Mariotti (2007) proposed *Rational Shortlisting Methods* as a model of human choice, which lead to number of works on shortlisting as a decision procedure, for example the works of Dutta and Horan (2015), Horan (2016), Kops (2018), and Tyson (2013).

More generally, there is a substantial literature on multi-winner voting with a *fixed* number of winners (i.e., committee size), as witnessed by recent surveys (Kilgour and Marshall 2012; Faliszewski et al. 2017; Lackner and Skowron 2023). Multi-winner voting rules are much better understood, both from an axiomatic (Elkind et al. 2017b; Fernández et al. 2017; Aziz et al. 2017a; Lackner and Skowron 2021; Sánchez-Fernández and Fisteus 2019) and experimental (Elkind et al. 2017a; Bredereck et al. 2019) point of view, also in the context of shortlisting (Aziz et al. 2017b; Bredereck et al. 2017). Results for multi-winner rules, however, typically do not easily translate to the setting with a variable number of winners.

## The Formal model

In this section we describe our formal model that embeds shortlisting in a voting framework. The model consists of two parts: a general framework for approval-based elections with a variable number of winners (Kilgour 2010, 2016; Faliszewski et al. 2020) on the one hand and, on the other hand, four basic axioms that we consider essential prerequisites for shortlisting rules.

An approval-based election $E =(C,V)$ consists of a non-empty set of candidates (or alternatives)^3^$C = \{c_1,\dots , c_m\}$ and an *n*-tuple of approval ballots $V =(v_1,\dots ,v_n)$ where $v_i \subseteq C$. If $c_j \in v_i$, we say that voter *i* approves candidate $c_j$; if $c_j \not \in v_i$, voter *i* does not approve candidate $c_j$. We interpret a voter’s approval of a candidate as the preference for this candidate being included in the shortlist. In the following we will always write $n_E$ for the number of voters and $m_E$ for the number candidates in an election *E*. We will omit the subscript if *E* is clear from the context.

The *approval score* $$ sc _E(c_j)$$ of candidate $$c_j$$ in election *E* is the number of approvals of $$c_j$$ in *V*, i.e., $$ sc _E(c_j)=|\{i: 1\le i \le n \text { and } c_j\in v_i\}|$$. We write $$ sc (E)$$ for the vector $$( sc _E(c_1), \dots $$, $$ sc _E(c_m))$$. To avoid unnecessary case distinctions, we only consider *non-degenerate* elections: these are elections where not all candidates have the same approval score.

An *approval-based variable multi-winner rule* (which we refer to just as “voting rule”) is a function mapping an election $E=(C,V)$ to a subset of *C*. Given a rule $\mathcal {R}$ and an election *E*, $\mathcal {R}(E)\subseteq C$ is the *winner set* according to voting rule $\mathcal {R}$, i.e., $\mathcal {R}(E)$ is the set of candidates which have been shortlisted. We refer to candidates in $\mathcal {R}(E)$ as *winners* or *winning candidates*. Note that $\mathcal {R}(E)$ may be empty or contain all candidates. We allow this because several of the shortlisting rules that are used in practice or have been proposed in the literature can lead to such winner sets. Additionally, in some applications, it might be preferable that a shortlisting rule fails by returning no/all candidates instead of returning a shortlist that contains bad candidates/excludes good candidates. For example, when selecting candidates for an interview, it might be better to know that there are no suitable candidates instead of interviewing candidates that do not fit the position. That being said, in most situations, the possibility of returning the empty set is very undesirable. This will be formalized as an axiom in Sect. 4.4.

Now we introduce the basic axioms that we require every shortlisting rule to satisfy. Anonymity and Neutrality are two basic fairness axioms for voting rules (Zwicker and Moulin 2016).

### Axiom 1

(Anonymity) All voters are treated equal, i.e., for every permutation $\pi : \{1,\dots ,n\}\rightarrow \{1,\dots ,n\}$ and election $E=(C,V)$ where $V= (v_1, \dots , v_n)$, if $E^*=(C,V^*)$ with $V^* = (v_{\pi (1)},\dots ,v_{\pi (n)})$, then $\mathcal {R}(E) = \mathcal {R}(E^*)$.

### Axiom 2

(Neutrality) All candidates are treated equally, i.e., for every election $E =(C,V)$ where $V= (v_1, \dots , v_n)$ and permutation $\pi : C\rightarrow C$, if $E^* = (C, V^*)$ where $V^* =(v_1^*,\dots ,v_n^*)$ with $v_i^* = \{\pi (c) \mid c \in v_i\}$, then $\pi (c) \in \mathcal {R}(E^*)$ iff $c \in \mathcal {R}(E)$ for all $c\in C$.

Shortlisting differs from other multi-winner scenarios in that we are not interested in representative or proportional committees. Instead, the goal is to select the most excellent candidates. This goal is formalized in the following axiom.

### Axiom 3

(Efficiency) No winner set can have a strictly smaller approval score than a non-winner, i.e., for all elections $$E=(C,V)$$ and all candidates $$c_i, c_j \in C$$ if $$ sc _E(c_i) > sc _E(c_j)$$ and $$c_j \in \mathcal {R}(E)$$ then also $$c_i \in \mathcal {R}(E)$$.

The assumption that approval scores are approximate measures of the general quality of candidates can also be argued in a probabilistic framework: under reasonable assumptions the set of candidates with the highest approval scores contains the maximum likelihood estimate of the truly best candidates (Procaccia and Shah 2015). Additionally, the accuracy of Approval Voting remains close to that of the maximum likelihood estimator even in situations where it is not optimal. Finally, directly computing the likelihood of a candidate being the (objectively) best, requires learning a potentially large number of probability values (Procaccia and Shah 2015). So, while this approach can be very useful in settings such as crowd-sourcing, we view it as less applicable in our voting-based shortlisting scenario, mainly due to the requirement of having (rather) simple, comprehensible aggregation methods.

Further work that considers learning a ground truth from approval ballots was done by Allouche et al. (2022a, b), who showed that when additional information is available, it can be used to achieve a higher accuracy. Allouche et al. (2022a) considered the case where the mechanism designer has an estimate of the size of the objectively best winner set. Allouche et al. (2022b) studied the case where the mechanism designer has reasons to believe that smaller approval ballots corresponds to higher certainty. Both assumptions are reasonable in some—but not all—shortlisting scenarios. Further, we note that these approaches do not fit in our framework as they are not based on approval scores. Studying whether the results of Allouche et al. (2022a, b) can be used to define better shortlisting rules when they are applicable is an important research direction for future work.

Efficiency can also be argued for from the perspective of voters: Let $\mathcal {R}$ satisfy Efficiency and $W=\mathcal {R}(E)$ for some election *E*. Then we claim that there does not exist a set $W'$ with $|W|=|W'|$ such that (i) $|W'\cap v|\ge |W\cap v|$ for all $v\in V$ and (ii) $|W'\cap w|> |W\cap w|$ for some $w\in V$. Otherwise $\sum _{c\in W'} sc _E(c) > \sum _{c\in W} sc _E(c)$ would hold. As $|W|= |W'|$ this implies that there is at least one candidate $c \in W' {\setminus } W$ with $sc _E(c) > \min \{ sc _E{c'} \mid c' \in W\}$, a contradiction. In this sense, efficient shortlists are *Pareto efficient among shortlists of the same size*.

It is also worth noting that Efficiency rules out proportional voting rules. It is easy to see why: a proportional selection of winner sets has to contain candidates supported by (sufficiently sized) minorities. As Efficiency demands that majority candidates are always to be preferred, any sensible notion of proportionality clashes with Efficiency.

The last of our basic axioms is Non-tiebreaking. Since the number of winners is variable in our setting, there is generally no need to break ties. Because tiebreaking is usually an arbitrary and unfair process, voting rules should not introduce unnecessary tiebreaking. This idea yields our fourth axiom:

### Axiom 4

(Non-tiebreaking) If two candidates have the same approval score, either both or neither should be winners. That is, for all elections $$E=(C,V)$$ and all candidates $$c_i$$ and $$c_j$$ if $$ sc _E(c_i) = sc _E(c_j)$$ then either $$c_i,c_j \in \mathcal {R}(E)$$ or $$c_i,c_j \not \in \mathcal {R}(E)$$.

We postulate these four axioms as the minimal requirements for a voting rule to be considered a shortlisting rule in our sense.

### Definition 1

An approval-based variable multi-winner rule is a shortlisting rule if it satisfies Anonymity, Neutrality, Efficiency and is non-tiebreaking.

Observe that Non-tiebreaking and Efficiency are axioms that are only interesting if we consider voting with a variable number of winners. Clearly, no voting rule for voting with a fixed number of winners can be non-tiebreaking. Furthermore, except for the issue of how to break ties, there is exactly one voting rule for approval voting with a fixed number *k* of winners that satisfies Efficiency, namely picking the *k* candidates with maximum approval score (*Multi-winner Approval Voting*).

A consequence of Efficiency and Non-tiebreaking is that a shortlisting rule only has to decide how many winners there should be.

This has two important consequences. First of all, nearly all results about shortlisting rules in this paper also apply in situations where only the approval score is known and not the full approval profile. These results are, therefore, also applicable if weighted approval voting is used or the scores of candidates are determined by other means than voting. However, some results presented in this paper apply also to more general classes of multi-winner voting rules and some rules and axioms can be more naturally formulated by referencing the set of voters. Therefore, we still assume that the full approval profile is known.

Second, requiring Efficiency and Non-tiebreaking reduces the complexity of finding the winner set drastically as there are only linearly many possible winner sets, in contrast to the exponentially many subsets of *C*.

### Observation 1

For every election $E=(C,V)$ there are at most $|C|+1$ sets that can be winner sets under a shortlisting rule.

## Shortlisting rules

In the following, we define the shortlisting rules that we study in this paper. We define these rules by specifying which properties a candidate has to satisfy to be contained in the winner sets. As before, let $E=(C,V)$ be an election. We assume additionally that $c_1, \dots , c_m$ is an enumeration of the candidates in descending order of approval score, i.e., such that $sc _E(c_{i-1}) \ge sc _E(c_i)$ for all $2 \le i \le m$. We will illustrate all rules on the following example:

### Example 1

Let $$E = (C,V)$$ be an election with 10 voters and 8 candidates $$c_1, \dots , c_8$$. The scores are given by $$ sc (E) = (10,10,9,8,6,3,3,0)$$. They are also repeated in Table 1.

**Table 1:** Scores from Example 1

	$c_1$	$c_2$	$c_3$	$c_4$	$c_5$	$c_6$	$c_7$	$c_8$
$sc _E(\cdot )$	10	10	9	8	6	3	3	0

There are seven possible winner sets for a shortlisting rule: $\emptyset$, $\{c_1,c_2\}$, $\{c_1, c_2, c_3\}$, $\{c_1, c_2, c_3, c_4\}$, $\{c_1, c_2, \ldots , c_5\}$, $\{c_1, c_2, \ldots , c_7\}$, $\{c_1, c_2, \ldots , c_8\}$.

### Established rules

First we introduce the shortlisting rules that are either commonly used in practice or have been proposed in the literature before. A natural idea is to select all most-approved candidates. The corresponding winner set equals the set of co-winners of classical Approval Voting (Brams and Fishburn 1978).

#### Rule 1

(Approval Voting) A candidate *c* is a winner iff *c*’s approval score is maximal, i.e., $c \in \mathcal {R}(E)$ iff $sc _E(c) = \max ( sc (E))$.

The winners under *Approval Voting* in Example 1 are $c_1$ and $c_2$ as they both have the highest score.

Another natural way to determine the winner set is to fix some percentage threshold and declaring all alternatives to be winners that surpass this approval threshold (Kilgour 2010). For example, for a baseball player to be entered into the Hall of Fame, more than 75% of the members of the Baseball Writers’ Association of America have to approve this nomination (BWAA 2019). Such rules are known as quota rules in judgment aggregation (Endriss 2016).

#### Rule 2

(*f*-Threshold) Let $$f:\mathbb {N} \rightarrow \mathbb {N}$$ be a function such that $$0< f(|V|) < |V|$$. Then, $$c \in \mathcal {R}(E)$$ for an alternative $$c \in C$$ if and only if $$ sc _E(c) > f(|V|)$$. We write $$\alpha $$-Threshold for a constant $$0\le \alpha < 1$$ to denote the *f*-Threshold rule with $$f(n)=\lfloor \alpha \cdot n\rfloor $$.

Consider for example $f(|V|) = \frac{|V|}{2}$. Then an alternative is a winner if it is approved by more than 50% of all voters. In Example 1 this would mean that the winner set contains all candidates with 6 or more approvals, i.e., $c_1, \ldots , c_5$.

A sensible modification of $f$-*Threshold* would be to select all alternatives with an above-average approval score, i.e., the set of winners consists of all alternatives *c* with $sc _E(c)>\frac{1}{m}\cdot \sum _{c'\in C} sc _E(c')$. This rule is also a shortlisting rule in our sense. However, as it will, in expectation, select half of the available candidates, we do not think that it is a reasonable rule in most shortlisting settings. Therefore, we do not study it and only mention that it might be a good rule in other voting contexts with a variable number of winners. For example, Duddy et al. (2016) analyzed this rule and concluded that it is the best rule for partitioning alternatives into homogeneous groups [see also the axiomatic characterization of this rule by Brandl and Peters (2019)].

Another natural modification is to base the threshold not on the number of voters but on the highest approval score achieved by a candidate. We call this *Max-Score*-$f$-*Threshold*. This variant of $f$-*Threshold* turns out to be well suited to shortlisting as it formalizes the goal of selecting all candidates that are close to the top.

#### Rule 3

(Max-Score-*f*-Threshold) Let $f:\mathbb {N} \rightarrow \mathbb {N}$ be a function such that $0< f(x) < x$. Then, $c \in \mathcal {R}(E)$ for an alternative $c \in C$ if and only if $sc _E(c) > f(\max sc (E))$. We write Max-Score$\alpha$-Threshold for a constant $0\le \alpha < 1$ to denote the Max-Score-*f*-Threshold rule with $f(n)=\lfloor \alpha \cdot n\rfloor$.

We observe that $c_1$ and $c_2$ in Example 1 have score *n*, hence $f$-*Threshold* and *Max-Score*-$f$-*Threshold* coincide on the example.

The next three rules are further shortlisting methods that have been proposed in the literature. *First Majority* (Kilgour 2016) includes as many alternatives as necessary to comprise more than half of all approvals. The following definition deviates slightly from the original definition (Kilgour 2016) in that it is non-tiebreaking.

#### Rule 4

(First Majority) Let *i* be the smallest index such that $\sum _{j \le i} sc _E(c_j)> \sum _{j > i} sc _E(c_j)$. Then $c \in \mathcal {R}(E)$ if and only if $sc _E(c)\ge sc _E(c_i)$.

The candidates in Example 1 together have 49 approvals. Therefore, a shortlist needs at least 25 approvals to be the First Majority winner set. The smallest shortlist to achieve at least 25 approvals is $\{c_1, c_2,c_3\}$ with 29 approvals.

Next-*k* (Brams and Kilgour 2012) is a rule that includes alternatives starting with the highest approval score, until a major drop in the approval scores is encountered, more precisely, if the total approval score of the next *k* alternatives is less than the score of the previous alternative.

#### Rule 5

(Next-*k*) Let *k* be a positive integer. Then, $$c_i \in \mathcal {R}(E)$$ if for all $$i' < i$$ it holds that $$ sc _E(c_{i'}) \le \sum _{j=1}^{k} sc _E(c_{i'+j})$$, where $$ sc _E(c_{i' +j}) = 0$$ if $$i' +j> m$$.

Consider Next-2. Then it is easy to check that, in Example 1, for all $i \le 5$ the score of $c_i$ is smaller or equal the sum of the scores of the next two candidates. For example $sc _E(c_1) = 10 \le 19 = sc _E(c_2) + sc _E(c_3)$. On the other hand $sc _E(c_7) = 3 \le 0 = sc _E(c_8) + 0$. Therefore, the winner set under Next-2 is $\{c_1, \dots , c_7\}$.

Observe that for both *Next*-$k$ and *First Majority* the winner set does not depend on the chosen enumeration of alternatives. This will hold for all voting rules introduced in this paper.

Faliszewski et al. (2020) discuss several other rules that satisfy our basic axioms called Capped Satisfaction Approval Voting (CSA), Net Approval Voting (NAV) and Net Capped Satisfaction Approval Voting (NCSA) which were originally proposed by Brams and Kilgour (2012, 2015) as well as generalizations of these rules. Among these, Faliszewski et al. (2020) conclude that only the following generalization of NCSA is practical.

#### Rule 6

(q-NCSA) Let $q \in [0,1]$ be a real number and $S \subseteq C$ a set of candidates. Then we define the *q-NCSA*-score of *S* as^4^:

$$\begin{aligned} sc _E^{\textit{q-NCSA}}(S)=\frac{1}{|S|^q}\sum _{c \in S}(2 sc _E(c) - n).\end{aligned}$$

The winner set then is the largest set with a maximum *q-NCSA*-score.^5^

It is not immediately obvious that *q-NCSA* is a shortlisting rule in our sense. The following proposition shows that this is indeed the case *q-NCSA*.

#### Proposition 1

The *q*-NCSA rule has the following properties for all $q \in [0,1]$.

1. *q*-NCSA is a shortlisting method.
2. *q*-NCSA can be computed in polynomial time.

#### Proof

We prove the both statements separately:

1. It is clear that *q-NCSA* satisfies Anonymity and Neutrality. Consider Efficiency: Assume there are two candidates $$c_i$$ and $$c_j$$ such that $$ sc _E(c_i) < sc _E(c_j)$$, $$c_i \in R(E)$$ and $$c_j \not \in R(E)$$. Then there must be a $$S \subseteq C$$ with $$c_i \in S$$ and $$c_j \not \in S$$ which has maximal *q-NCSA*-score. However, by definition the *q-NCSA*-score of $$(S\setminus \{c_1\}) \cup \{c_j\}$$ is higher than the *q-NCSA*-score of *S*. A contradiction. The non-tiebreaking property follows from the following claim:

#### Claim 1

If $$ sc _E(c_i) = sc _E(c_{i+1})$$ and

$$\begin{aligned} sc _E^{\textit{q-NCSA}}(\{c_1, \ldots , c_{i-1}\}) \le sc _E^{\textit{q-NCSA}}(\{c_1, \ldots , c_{i}\}),\end{aligned}$$

then also

$$\begin{aligned} sc _E^{\textit{q-NCSA}}(\{c_1, \ldots , c_{i}\}) \le sc _E^{\textit{q-NCSA}}(\{c_1, \dots , c_{i+1}\}).\end{aligned}$$

By Efficiency, the largest set with maximum *q-NCSA*-score is of the form $$\{c_1, \ldots ,c_i\}$$ for some $$c_i$$. As the set has maximum *q-NCSA*-score, it holds in particular that $$ sc _E^{\textit{q-NCSA}}(\{c_1, \ldots , c_{i-1}\}) \le sc _E^{\textit{q-NCSA}}(\{c_1, \ldots , c_{i}\})$$. If $$ sc _E(c_i) = sc _E(c_{i+1})$$, i.e., if $$\{c_1, \ldots ,c_i\}$$ breaks a tie, then it follows from the claim that $$ sc _E^{\textit{q-NCSA}}(\{c_1, \ldots , c_{i}\}) \le sc _E^{\textit{q-NCSA}}(\{c_1, \ldots , c_{i+1}\})$$. However, this is a contradiction to the assumption that $$\{c_1,\ldots ,c_i\}$$ was the largest set with maximal *q-NCSA*-score. The proof of Claim 1 contains a lengthy calculation and can be found in the Appendix.

2. As we have shown that *q-NCSA* is a shortlisting rules, we know that we only need to consider sets that are efficient and non-tiebreaking. Further, we can clearly compute the *q-NCSA*-score of a set in polynomial time. As there are at most linearly many potential winner sets (Observation 1), finding the one with maximum *q-NCSA*-score can be done in polynomial time.

$$\square $$

Consider again Example 1. Then, the 0.5-NCSA-score of the shortlist $\{c_1, \dots , c_4\}$ is $({(2\cdot 10 -10) + (2\cdot 10 -10) + (2\cdot 9 -10) + (2\cdot 8 -10)})/{\sqrt{4}} = 17.$ It can be checked that this is the unique maximal 0.5-NCSA-score and hence $\{c_1, \dots , c_4\}$ is the winner set under 0.5-*NCSA*.

#### Observation 2

An important feature (and downside) of *q-NCSA* is that candidates with an approval score of less than *n*/2 can only decrease $sc _E^{\textit{q-NCSA}}(S)$. Consequently, *q-NCSA* returns the empty in elections where all candidates have few approvals.

### New shortlisting rules

Let us now introduce some new shortlisting rules. Similarly to *Next*-$k$, the next two rules are based on the idea that one wants to make the cut between winners and non-winners in a place where there is a large gap in the approval scores. This can either be the overall largest gap or the first sufficiently large gap.

#### Rule 7

(Largest Gap) Let *i* be the smallest index such that $sc _E(c_{i}) - sc _E(c_{i+1}) = \max _{j < m} ( sc _E(c_j) - sc _E(c_{j+1}))$. Then $c \in \mathcal {R}(E)$ if and only if $sc _E(c)\ge sc _E(c_i)$.

Note that in this definition a smallest index is guaranteed to exist due to our assumption that profiles are non-degenerate. In Example 1 the two largest gaps are between $c_5$ and $c_6$ and $c_7$ and $c_8$, both of size 3. As we pick the smaller index, the winner set is $\{c_1, \dots , c_5\}$.

#### Rule 8

(First *k*-Gap) Let *i* be the smallest index such that $sc _E(c_i) - sc _E(c_{i+1}) \ge k$. Then $c \in \mathcal {R}(E)$ if and only if $sc _E(c)\ge sc _E(c_i)$. If no such index exists, then $\mathcal {R}(E) = C$, i.e., every alternative is a winner.

Let us consider *First 2-Gap* in Example 1. The gaps between $c_1$ and $c_2$, $c_2$ and $c_3$ as well as between $c_3$ and $c_4$ are smaller than two, while the gap between $c_4$ and $c_5$ is 2. Therefore the winner set is $\{c_1,c_2, c_3, c_4\}$.

The parameter *k* has to capture what it means in a given shortlisting scenario that there is a sufficiently large gap between alternatives, which in particular depends on the number of voters $|V|$. If no further information is available, one can choose *k* by a simple probabilistic argument. Assume, for example, alternative *c*’s approval score is binomially distributed $sc _E(c)\sim B(n, q_c)$, where *n* is the number of voters and $q_c$ can be seen as *c*’s quality. We choose *k* such that the probability of events of the following type are smaller than a selected threshold $\alpha$: two alternatives *a* and *b* have the same objective quality ($q_a=q_b$) but have a difference in their approval scores of *k* or more. In such a case, the *First* $k$-*Gap* rule might choose one alternative and not the other even though they are equally qualified, which is an undesirable outcome. For example, if $n= 100$ and we want $\alpha = 0.5$, we have to choose $k \ge 5$ and if we want $\alpha = 0.1$ we need $k \ge 12$. Note that this argument leads to rather large *k*-values; if further assumptions about the distribution of voters can be made, smaller *k*-values are feasible.

The voting rules above output winner sets of very different sizes (as we will see in the experimental evaluation, Sect. 5). It is a common case, however, that there is a preferred size for the winner set, but this size can be varied in order to avoid tiebreaking. This flexibility is especially crucial if the electorate is small and ties are more frequent. Based on real-world shortlisting processes, we propose a rule that deals with this scenario by accepting a preference order over set sizes as parameter and selecting a winner set with the most preferred size that does not require tiebreaking.

#### Rule 9

(Size Priority) Let $\vartriangleright$ be a strict total order on $\{0, \dots , m\}$, the *priority order*. Then $\mathcal {R}(E)=\{c_i\in C \mid 1\le i \le k\}$ if and only if

- either $sc _E(c_{k}) \ne sc _E(c_{k +1})$ or $k = 0$ or $k = m$,
- and $sc _E(c_{\ell }) = sc _E(c_{\ell +1})$ for all $\ell \vartriangleright k$.

Consider for example a strict total order of the form $$1 \vartriangleright 6 \vartriangleright 0 \vartriangleright \dots $$. Then the set of *Size Priority* winners under $$\vartriangleright $$ in Example 1 is the empty set, because $$\{c_1\}$$ and $$\{c_1, \dots , c_6\}$$ break ties, as $$ sc _E(c_1) = sc _E(c_2)$$ and $$ sc _E(c_6) = sc _E(c_7)$$.

*Size Priority* is a non-tiebreaking analogue of *Multi-winner Approval Voting*, which selects the *k* alternatives with the highest approval score. A specific instance of *Size Priority* was used by the Hugo Award prior to 2017 with the priority order $5 \vartriangleright 6 \vartriangleright 7 \dots$ (The Hugo Awards 2019). Generally, the choice of a priority order depends on the situation at hand. For award-shortlisting, typically a small number of alternatives is selected (the Booker Prize, e.g., has a shortlist of size 6). In a much more principled fashion, Amegashie (1999) argues that the optimal size of the winner set for shortlisting should be proportional to $\sqrt{m}$, i.e., the square root of the number of alternatives.

Now, how does a preferred size for the shortlist translate to a priority order? One possibility would be to order the possible sizes of the shortlist according to their distance to the most preferred size, breaking ties in some way, for example $k \vartriangleright k+1 \vartriangleright k-1 \vartriangleright k+2 \vartriangleright \cdots$. In practice, the most common priority order is $k \vartriangleright k+1 \vartriangleright \dots \vartriangleright m$ for some $k < m$, i.e., the smallest non-tiebreaking shortlist that contains at least *k* alternatives is selected. Another important special case are instances of *Size Priority* that rank 0 and *m* the lowest, i.e., that avoid return all or no candidates whenever possible. Therefore, we give *Size Priority* rules based on such priority orders a special name.

#### Definition 2

Let $\vartriangleright$ be a strict total order on $0, \dots , m$ and let *k* be a positive integer with $k \le m$ such that $k \vartriangleright k+1 \vartriangleright \cdots \vartriangleright m$ and $m \vartriangleright \ell$ for all $\ell < k$. Then, the *Size Priority* rule defined by the priority order $\vartriangleright$ is an *Increasing Size Priority* rule. We will write *ISP-k* as a short form for the *Increasing Size Priority* rule with $k \vartriangleright k+1 \vartriangleright \dots$ as priority order.

Let $\vartriangleright$ be a strict total order on $0, \dots , m$ such that $k \vartriangleright m$ and $k \vartriangleright 0$ holds for all $0< k < m$. Then, the *Size Priority* rule defined by the priority order $\vartriangleright$ is a *Decisive Size Priority* rule.

Other special cases of *Size Priority* could be defined in a similar way, for example *Decreasing Size Priority*. However, *Increasing Size Priority* and *Decisive Size Priority* are the most natural and common types of *Size Priority* and additionally satisfies better axiomatic properties than, e.g., *Decreasing Size Priority*.

Finally, we propose a rule that combines the ideas behind *First* $k$-*Gap* and *Size Priority*. In practice, we often want to have a large gap between winners and non-winners, but not at any price in terms of the size of the shortlist.

#### Rule 10

(Top-*s*-First-*k*-gap) Let $W'$ be the winner set for *First* $k$-*Gap* and $W''$ the winner set for the *Increasing Size Priority* instance defined by the order $s \vartriangleright s+1 \vartriangleright \dots$ (*ISP-s*). If $|W'|\le s$, return $W'$. Otherwise return $W''$.

Consider, for example, *Top*-$3$-*First*-$2$-*Gap*. Then, in Example 1 we know that $W' = \{c_1, \dots , c_4\}$ is the *First-2-gap* winner set. On the other hand, the shortlist $W'' = \{c_1,c_2,c_3\}$ is non-tiebreaking and therefore the *Size Priority* winner set for $3 \vartriangleright 4 \vartriangleright \dots$. As $|W'|> s$, the winner set under $\textit{Top}-$3$-\textit{First}-$2$-\textit{Gap}$ is $W''$.

Let us now consider the relationships between the proposed rules.

#### Proposition 2

We observe the following relations between the considered voting rules:

- *First* $k$-*Gap* and *Next*-$k$ are equivalent to *Approval Voting* for $k =1$.
- *ISP-1* is equivalent to *Approval Voting*.
- *Top*-$s$-*First*-$k$-*Gap* is equivalent to *First* $k$-*Gap* for $s = m$ and it is equivalent to *Increasing Size Priority* for $k = m$.

#### Proof

First observe that *First-1-Gap* and *Next-1* select all candidates which have maximal score. Now let $c_i$ be the first candidate which has less than the maximal score. Then $sc _E(c_{i-1}) - sc _E(c_i) \ge 1$ and thus *First-1-Gap* selects $\{c_1,\dots ,c_{i-1}\}$ (as does *Approval Voting*). Further, $sc _E(c_{i-1} > sc _E(c_i) = \sum _{j=1}^1 sc _E(c_{i-1+j})$ and thus *Next-1* selects $\{c_1,\dots ,c_{i-1}\}$. The argument for *ISP-1* is similar.

Finally, consider *Top*-$s$-*First*-$k$-*Gap*. If $s = m$, then $W'' = C$. Consequently, $|W'|\le |W''|$ and $W'$ is thus the winner set. On the other hand, if $k = m$ then $W' = C$. Hence, $|W''|\le |W'|$ and $W''$ is thus the winner set. $\square$

We finally observe that *q-NCSA* for $q=1$ is a mix of *Approval Voting* and $f$-*Threshold* and for $q= 0$ is closely related to $f$-*Threshold* for $f(n) = \frac{1}{2}n$. First consider $q= 1$. If any candidate is approved by more than $50\%$ of the voters then *1-NCSA* is equivalent to *Approval Voting*, as the *1-NCSA*-score equals the average net-approval of the candidates in the set. This score is maximized by any set only containing candidates with maximal approval. On the other hand, if no candidate has more than $50\%$ approvals then no set has positive *q-NCSA*-score. Therefore, the empty set is the smallest set with maximal *q-NCSA*-score.

Now consider $$q=0$$. We observe that then *q-NCSA*-score of a set $$S \subseteq C$$ is the sum of the *net-approval* of the candidates, where the net approval of a candidate *c* is $$ sc _E(c) - (n - sc _E(c))$$. Hence the *0-NCSA*-score is maximized by every set that contains all candidates with positive net approval and an arbitrary number of candidates with 0 net approval. A candidate has non-negative net approval if and only if $$2 sc _E(c) -n \ge 0$$ which is equivalent to $$ sc _E(c) \ge \frac{n}{2}$$.^6^

To conclude the section, let us remark that all of the above rules can be computed in polynomial time. This follows immediately from their respective definitions. For *q-NCSA*, we made the argument explicit in Proposition 1.

## Axiomatic analysis

In this section, we axiomatically analyze shortlisting rules with the goal to discern their defining properties. First, we consider axioms that are motivated by the specific requirements of shortlisting, then we study well-known axioms that describe more generally desirable properties of voting rules. For an overview, see Table 2.

The proofs and counter-examples showing for each shortlisting rule whether it satisfies a specific axiom can be found in Appendix B.

**Table 2:** Results of the axiomatic analysis

	Unanimity	Anti-unanimity	$\ell$-Stability	Determined	Independence	Ind. of losing alt.	Res. to clones	Set monot.	Superset monot.
Approval voting	$\checkmark$	$\checkmark$	$\times$	$\checkmark$	$\times$	$\checkmark$	$\checkmark$	$\checkmark$	$\checkmark$
*f*-Threshold	$\checkmark$	$\checkmark$	$\times$	$\times$	$\checkmark$	$\checkmark$	$\checkmark$	$\checkmark$	$\times$
Max-Score-*f*-Threshold	$\checkmark$	$\checkmark$	$\times$	$\checkmark$	$\times$	$\checkmark$	$\checkmark$	$\checkmark$	$\times$
First majority	$\checkmark$	$\checkmark$	$\times$	$\checkmark$	$\times$	$\times$	$\times$	$\times$	$\times$
*q*-NCSA	$\checkmark$	$\checkmark$	$\times$	$\times$	$\times$	$\checkmark$	$\times$	$\checkmark$	$\times$
Next-*k*	$\checkmark$	$\checkmark$	$\times$	$\checkmark$	$\times$	$\times$	$\times$	$\checkmark$	$\times$
Largest gap	$\checkmark$	$\checkmark$	$\times$	$\checkmark$	$\times$	$\times$	$\checkmark$	$\checkmark$	$\times$
First *k*-gap	$\checkmark$	$\times$	$\ell \le k$	$\checkmark$	$\times$	$\checkmark$	$\checkmark$	$\checkmark$	$\checkmark$
Decis. size priority	$\checkmark$	$\checkmark$	$\times$	$\checkmark$	$\times$	$\times$	$\times$	$\checkmark$	$\times$
Incr. size priority	$\checkmark$	$\times$	$\times$	$\checkmark$	$\times$	$\checkmark$	$\times$	$\checkmark$	$\checkmark$
Top-*s*-First-*k*-Gap	$\checkmark$	$\times$	$\times$	$\checkmark$	$\times$	$\checkmark$	$\times$	$\checkmark$	$\checkmark$

### $\ell$-Stability, unanimity, and anti-unanimity

When shortlisting is used for the initial screening of candidates, for example for an award or a job interview, then we cannot assume that the voters have perfect judgment. Otherwise, there would be no need for a second round of deliberation, as we could just choose the highest-scoring alternative as a winner. Therefore, small differences in approval may not correctly reflect which alternative is more deserving of a spot on the shortlist. Thus, out of fairness, we want our voting rule to treat alternatives differently only if there is a significant difference in approval between them.

#### Axiom 5

($\ell$-Stability) If the approval scores of two alternatives differ by less than $\ell$, either both or neither should be a winner, i.e., for every election $E=(C,V)$ and candidates $c_i$ and $c_j$ if $| sc _E(c_i) - sc _E(c_j)|< \ell$ then either $c_i,c_j \in \mathcal {R}(E)$ or $c_i,c_j \not \in \mathcal {R}(E)$.

Here, the parameter $\ell$ has to capture what constitutes a significant difference in a given election. This will depend, for example, on the number and trustworthiness of the voters. Also, observe that 1-Stability equals Non-tiebreaking. Hence, we are only interested in $\ell$-Stability for $\ell \ge 2$.

Now, while a small difference in approvals might not correctly reflect the relative quality of the candidates, we generally assume in shortlisting that the approval scores approximate the underlying quality of alternatives.^7^ Therefore, at a minimum, we want to include alternatives that are approved by everyone and exclude alternatives that are approved by no one.

#### Axiom 6

(Unanimity) If an alternative is approved by everyone, it must be a winner, i.e., for every election $$E=(C,V)$$, if $$ sc _E(c) = n$$ then $$c \in \mathcal {R}(E)$$.

#### Axiom 7

(Anti-Unanimity) If an alternative is approved by no one, it cannot win, i.e., for every election $$E=(C,V)$$ if $$ sc _E(c) = 0$$ then $$c \not \in \mathcal {R}(E)$$.

Unfortunately, it turns out that these three axioms are incompatible unless there are many more voters than alternatives. Indeed Unanimity, Anti-Unanimity and $\ell$-Stability can be jointly satisfied if and only if $|V|\ge l\cdot |C|+1$.

#### Theorem 3

For every $\ell$ there is a shortlisting rule that satisfies Unanimity, Anti-Unanimity and $\ell$-Stability for every election *E* such that $n_E > (\ell - 1)\cdot (m_E -1)$. This is a tight bound in the following sense: For every $\ell >1$, there is an election *E* such that $n_E = (\ell - 1)\cdot (m_E -1)$ and no shortlisting rule can satisfy Unanimity, Anti-Unanimity and $\ell$-Stability on *E*.

#### Proof

To show that Unanimity, Anti-Unanimity and $$\ell $$-Stability are jointly satisfiable if $$n_E > (\ell - 1)\cdot (m_E -1)$$, we will show that a slightly modified version of *First* $$k$$-*Gap* satisfies all three axioms for elections *E* with $$n_E > (\ell -1) \cdot (m_E - 1)$$. We define *Modified First* $$\ell $$-*Gap* as follows: Let $$c_1, \dots , c_m$$ be an enumeration of *C* such that $$ sc _E(c_{i-1}) \ge sc _E(c_{i})$$. Let *i* be the smallest index such that $$ sc _E(c_i) - sc _E(c_{i+1}) \ge \ell $$. Then $$c \in \mathcal {R}(E)$$ if and only if $$ sc _E(c)\ge sc _E(c_i)$$. If no such index exists, then $$\mathcal {R}(E) = \emptyset $$ if there is an alternative *c* with $$ sc _E(c) = 0$$, and $$\mathcal {R}(E) = C$$ otherwise. Clearly, this rule still satisfies $$\ell $$-Stability.

Now, let *E* be an election such that there is an alternative *c* with $$ sc _E(c) = n$$. Assume first that there is no alternative $$c'$$ with $$ sc _E(c') = 0$$. In that case, *Modified First* $$\ell $$-*Gap* vacuously satisfies Anti-Unanimity and, by definition, also Unanimity. Now assume that there is an alternative *c* with $$ sc _E(c) = 0$$. We claim that there is an index *i* such that $$ sc _E(c_i) - sc _E(c_{i+1}) \ge \ell $$ and hence only alternatives *c* such that $$ sc _E(c) \ge sc _E(c_i)> \ell -1$$ are winners. Otherwise, we have $$ sc _E(c_{i+1}) \ge sc _E(c_{i}) - (\ell -1) $$ for all $$i < m$$ and hence $$ sc _E(c_m) \ge sc _E(c_{1}) -(\ell -1)\cdot (m-1)$$. However, as $$ sc _E(c_1) = n > (\ell -1)\cdot (m-1)$$ this contradicts the assumption that there is an alternative *c* with $$ sc _E(c) = 0$$, i.e., $$ sc _E(c_m)=0$$.

Finally, let *E* be an election such that there is no alternative *c* with $$ sc _E(c) = n$$. Then, *Modified First* $$\ell $$-*Gap* vacuously satisfies Unanimity. Now, if there is an alternative $$c'$$ with $$ sc _E(c') = 0$$ then we have to distinguish two cases. If there is no $$\ell $$-gap, then $$\mathcal {R}(E) = \emptyset $$ by definition and hence *Modified First* $$\ell $$-*Gap* satisfies Anti-Unanimity. On the other hand, if there is a $$\ell $$-gap, then only alternatives above the $$\ell $$-gap are selected, which must have a score of $$\ell $$ or larger. Hence, Anti-Unanimity is also satisfied.

Now we show the tightness of the theorem. Let *E* be an election with 2 alternatives and $\ell -1$ voters such that $sc (E) = (\ell -1,0)$. We observe $n_E = \ell -1 = (\ell -1)\cdot (2-1)$. We claim that no $\mathcal {R}$ satisfy Unanimity, Anti-Unanimity and $\ell$-Stability on *E*. Hence, $c_1 \in \mathcal {R}(E)$ must hold by Unanimity. Then $sc _E(c_1) - sc _E(c_2) < \ell$ implies $c_2 \in \mathcal {R}(E)$ by $\ell$-Stability, contradicting Anti-Unanimity. $\square$

Theorem 3 tells us that $\ell$-Stability requires some sacrifices as it is incompatible with the combination of Unanimity and Anti-Unanimity. However, *First* $k$-*Gap* can be seen as an optimal compromise as, with a small modification, it satisfies Anti-Unanimity whenever Theorem 3 allows it.

We observe that *First* $k$-*Gap* is the only voting rule considered in this paper that satisfies $\ell$-Stability for $\ell >1$: However, it is worth noting that *Largest Gap* satisfies $\ell$-Stability whenever there is an $\ell$-gap.

Next we will consider axioms that are not specific to shortlisting, but often appear in the voting and judgment aggregation literature to characterize “well behaved” aggregation techniques.

### Independence

$\ell$-Stability formalizes the idea that the length of a shortlist should take the magnitude of difference between approval scores into account. This contradicts an idea that is often considered in judgment aggregation, namely that all alternatives should be treated independently (Endriss 2016).

#### Axiom 8

(Independence) If an alternative is approved by exactly the same voters in two elections then it must be a winner either in both or in neither. That is, for an alternative *c*, and two elections $E = (C,V)$ and $E^* = (C,V^*)$ with $|V|= |V^*|$ and $c \in v_i$ if and only if $c \in v_i^*$ for all $i\le n$, it holds that $c \in \mathcal {R}(E)$ if and only if $c \in \mathcal {R}(E^*)$.

$f$-*Threshold* rules are the only rules in our paper satisfying Independence. Indeed, Independence characterizes $f$-*Threshold* rules, except for some edge cases that are not interesting in practice.

#### Theorem 4

Given a fixed set of alternatives *C*, every shortlisting rule that satisfies Independence is either a $f$-*Threshold* rule for some function *f*, always returns the empty set or always returns the full set *C*.

#### Proof

Let $$\mathcal {R}$$ be a voting rule that satisfies Anonymity and Independence. Then we claim that for two elections $$E = (C,V)$$ and $$E^* = (C,V^*)$$ with $$|V|= |V^*|$$ and an alternative $$c_i \in C$$ we have that $$ sc _E(c_i) = sc _{E^*}(c_i)$$ implies that either $$c_i \in \mathcal {R}(E),\mathcal {R}(E^*)$$ or $$c_i \not \in \mathcal {R}(E),\mathcal {R}(E^*)$$. If $$ sc _E(c_i) = sc _{E^*}(c_i)$$, then there is a permutation $$\pi : \{1,\dots ,n\} \rightarrow \{1,\dots ,n\}$$ such that $$c_i \in v_i$$ if and only if $$c_i \in v^*_{\pi (i)}$$. Now, let $$E' = (C, \pi (V))$$. Then, by Anonymity, $$c_i \in \mathcal {R}(E)$$ if and only if $$c_i \in \mathcal {R}(E')$$. Now, as $$c_i$$ is approved by the same voters in $$E'$$ and $$E^*$$, Independence implies $$c_i \in \mathcal {R}(E')$$ if and only if $$c_i \in \mathcal {R}(E^*)$$.

Now, let $$E = (C,V)$$ and $$E^* = (C,V^*)$$ be two elections with $$|V|= |V^*|$$. Furthermore, assume $$c_i \in \mathcal {R}(E)$$ and $$ sc _E(c_i) < sc _{E^*}(c_i)$$. We claim that this implies $$c_i \in \mathcal {R}(E^*)$$. By Independence, we can assume w.l.o.g. that there is an alternative $$c_j$$ such that $$ sc _E(c_j) = sc _{E^*}(c_i)$$. Then, by Efficiency, $$c_j \in \mathcal {R}(E^*)$$. Now, let $$E'$$ be the same election as *E* but with $$c_i$$ and $$c_j$$ switched. Then by Neutrality we have $$c_i \in \mathcal {R}(E')$$. As by definition $$ sc _{E'}(c_i) = sc _{E^*}(c_i)$$ this implies $$c_i \in \mathcal {R}(E^*)$$ by Anonymity and Independence.

The two arguments above mean that for every alternative $c_i$ and $n \in \mathbb {N}$ there is a *k* such that for all elections $E = (C,V)$ with $|V|= n$ we know $c_i \in \mathcal {R}(E)$ if and only if $sc _E(c_i) \ge k$. If $\mathcal {R}$ also satisfies Neutrality, then *k* must be the same for every $c_i \in C$. Finally, if we have $0 < k \ge n$, then $\mathcal {R}$ is a Threshold rule, if $k > n$, then $\mathcal {R}$ always returns the empty set and if $k = 0$, then $\mathcal {R}$ always returns *C*. $\square$

In light of Theorem 4, Independence seems to be a very strong requirement, therefore we also consider the axiom Independence of Losing Alternatives which can be seen as a weakening of Independence. It states that removing a non-winning alternative cannot change the outcome of an election.

#### Axiom 9

(Independence of Losing Alternatives) Let $E = (C,V)$ with $V=(v_1,\dots ,v_n)$ and $E^* = (C^*, V^*)$ where $C^* = C {\setminus } \{c^*\}$ and $V^*=(v_1^*,\dots ,v_n^*)$ be two elections such that $c^* \not \in \mathcal {R}(E)$ and $v_i^* = v_i {\setminus } \{c^*\}$ for all $i\le n$. Then $\mathcal {R}(E) = \mathcal {R}(E^*)$.

For *Size Priority* we encounter a difficulty: Independence of Losing Alternatives cannot be applied to *Size Priority* because each instance of *Size Priority* is defined by a linear order on $0, \dots , m$ and decreasing the number of alternatives necessitates a different order. We can deal with this problem by defining classes of *Size Priority* instances:

#### Definition 3

Let $\vartriangleright$ be a strict total order on $\mathbb {N}$. Then the class of *Size Priority* instances defined by $\vartriangleright$ contains for every number of alternatives *m* the *Size Priority* instance given by the restriction of $\vartriangleright$ to $\{0,1,\dots ,m\}$.

We say that the class of *Size Priority* instances defined by $\vartriangleright$ is a class of *Increasing Size Priority* instances if every *Size Priority* instance in the class is an *Increasing Size Priority* instance.

This definition allows us to ask whether classes of *Size Priority* instances (defined by $\vartriangleright$) satisfies Independence of Losing Alternatives.

### Stability of outcomes: resistance to clones and monotonicity

Next, we consider three classic axioms of social choice theory, namely Resistance to Clones (Tideman 1987) and two monotonicity axioms (Zwicker and Moulin 2016) adapted to the shortlisting setting. All three axioms formalize the idea that there are specific changes of the election instance that should not change the outcome.

First we consider Resistance to Clones. In many shortlisting scenarios, for example in the context of recommender systems, it is not always clear if alternatives should be bundled together. For example, if we want to select a number of books to recommend, should we include each part of a trilogy separately or bundle the whole series? Shortlisting rules that satisfy Resistance to Clones are useful because the outcome of the rule is the same in both cases (if all parts of the series are equally popular).

#### Axiom 10

(Resistance to Clones) Adding a clone of an alternative to an election does not change the outcome, i.e., if $E = (C,V)$ and $E^* = (C \cup \{c^*\}, V^*)$ are two elections with $|V|= |V^*|$ such that, for all $j \le n$, we have $c_i \in v_j$ if and only if $c_i \in v_j^*$ for all $c_i \in C$ and $c^* \in v_j^*$ if and only if $c_k \in v_j$ for some $k \le m$, then $\mathcal {R}(E) = \mathcal {R}(E^*)$ if $c_k \not \in \mathcal {R}(E)$ and $\mathcal {R}(E^*) = \mathcal {R}(E) \cup \{c^*\}$ if $c_k \in \mathcal {R}(E)$.

The first monotonicity axiom we consider is Set Monotonicity. It states that if one voter additionally approves the winner set, this must not change the outcome.

#### Axiom 11

(Set Monotonicity) For any two elections $E = (C,V)$ and $E^* = (C,V^*)$ with $V = (v_1, \dots , v_n)$ and $V^* = (v^*_1, \dots , v^*_n)$, if there exists a $j \le n$ such that $v_j \cap \mathcal {R}(E) = \emptyset$, $v^*_j = v_j \cup \mathcal {R}(E)$ and $v^*_l = v_l$ for all $l \ne j$, then $\mathcal {R}(E^*) = \mathcal {R}(E)$.

All of our rules except *First Majority* and *Max-Score*-$f$-*Threshold* with non-constant threshold function satisfy Set Monotonicity. Set Monotonicity is a very natural axiom for many applications, so the fact that *First Majority* does not satisfy it makes it hard to recommend the rule in most situations. We can strengthen this axiom as follows: a voter that previously disapproved all winning alternatives changes her mind and now approves a superset of all (previously) winning alternatives; this should not change the set of winning alternatives. This is a useful property as it guarantees that if an additional voter enters the election, who agrees with the set of currently winning alternatives but might approve additional alternatives, then the set of winning alternatives remains the same and, in particular, does not expand.

#### Axiom 12

(Superset Monotonicity) Let $E = (C,V = (v_1, \dots , v_n))$ be an election. If $E^* = (C,V^* = (v^*_1, \dots , v^*_n))$ is another election such that for some $j \le n$ we have $v_j \cap \mathcal {R}(E) = \emptyset$, $\mathcal {R}(E) \subseteq v^*_j$ and $v^*_l = v_l$ for all $l \ne j$, then $\mathcal {R}(E) = \mathcal {R}(E^*)$.

In contrast to Set Monotonicity, only few rules satisfy Superset Monotonicity. *Increasing Size Priority* satisfies Superset Monotonicity as any ties between winners remain. Moreover, as the size of the gap between winners and non-winners cannot decrease and gaps within the winner set remain, *First* $k$-*Gap* satisfies Superset Monotonicity for all *k* (which includes *Approval Voting*). For this reason *Top*-$s$-*First*-$k$-*Gap* also satisfies Superset Monotonicity by an analogous argument as for Set Monotonicity.

In general, the axioms discussed in this section can be seen as axioms about the stability of the winner set under specific changes to the election. We observed that *First Majority* and, to a lesser degree, *Size Priority* and *Next*-$k$ did not perform well in this regard. On the other hand, it seems that the winner set of *First* $k$-*Gap* and *Approval Voting* are particularly stable, as they are the only rule that satisfies all three axioms considered in this section.

### Minimal voting rules

The goal of shortlisting is to reduce a set of alternatives to a more manageable set of alternatives. It is therefore desirable that shortlisting rules produce short shortlists, without compromising on quality. To formalize this desideratum we define the concept of a minimal voting rule that satisfies a set of axioms.

#### Definition 4

Let $\mathcal {A}$ be a set of axioms and let $S(\mathcal {A})$ be the set of all voting rules satisfying all axioms in $\mathcal {A}$. Then, we say a voting rule is a minimal voting rule $\mathcal {R}$ for $\mathcal {A}$ if for all elections *E* it holds that $\mathcal {R}(E) = \bigcap _{\mathcal {R}^* \in S(\mathcal {A})} \mathcal {R}^*(E)$.

We observe that in general a minimal voting rule $\mathcal {R}$ for a set of axioms $\mathcal {A}$ does not satisfy all axioms in $\mathcal {A}$. Consider, e.g., the following axiom:

#### Axiom 13

(Determined) Every election must have at least one winner, i.e., for all elections *E* we have $\mathcal {R}(E) \ne \emptyset$.

Observe that, besides $f$-*Threshold*, *q-NCSA* and *Size Priority*, all voting rules considered in this paper are determined by definition.

Now, let us consider arbitrary voting rules with a variable number of winners, i.e., not only shortlisting rules. Then for every $c \in C$ the rule $\mathcal {R}_c$ that always outputs the set $\{c\}$ is a determined voting rule. It follows that the minimal determined voting rule always outputs the empty set and is hence not determined. In contrast, for shortlisting rules the following holds.

#### Proposition 5

Let $\mathcal {A}$ be a set of axioms that contains the four basic shortlisting axioms (Axioms 1–4). Then the minimal voting rule for $\mathcal {A}$ is again a shortlisting rule, i.e., it satisfies Axioms 1–4.

#### Proof

Let $\mathcal {A}$ be a set of axioms and let $\mathcal {R}$ be the minimal voting rule for $\mathcal {A}$. It is straightforward to see that $\mathcal {R}$ satisfies Neutrality and Anonymity. We show that $\mathcal {R}$ also satisfies Efficiency and is non-tiebreaking. Let *E* be an election. As every rule in $S(\mathcal {A})$ is a shortlisting rule, there is a $k_{\mathcal {R}^*} \in \{0,\dots , m\}$ for every rule $\mathcal {R}^* \in S(\mathcal {A})$ such that $\mathcal {R}^*(E) = \{c_1, \dots , c_{k_{\mathcal {R}^*}}\}$. Now let $k_m$ be the smallest *k* such that there is a rule $\mathcal {R}^* \in S(\mathcal {A})$ with $\mathcal {R}^*(E) = \{c_1, \dots , c_{k}\}$. Then, by definition $\mathcal {R}(E) = \mathcal {R}^*(E)$. As $\mathcal {R}^*(E)$ does not violate Efficiency and non-tiebreaking for *E*, neither does $\mathcal {R}$. As this argument holds for arbitrary elections, $\mathcal {R}$ satisfies Efficiency and is non-tiebreaking. $\square$

As the voting rule that always outputs the empty set is a shortlisting rule, it is also the minimal shortlisting rule (without additional axioms). Therefore, we need to assume additional axioms. We first look at determined and $\ell$-stable shortlisting rules.

#### Theorem 6

*Approval Voting* is the minimal voting rule that is efficient, non-tiebreaking and determined. Furthermore, for every positive integer *k*, *First* $k$-*Gap* is the minimal voting rule that is efficient, *k*-stable and determined.

#### Proof

Let $\mathcal {A}$ be the set $\{\text{ Efficiency }, k\text{-Stability }, \text{ Determined }\}$ and $\mathcal {R}$ be *First* $k$-*Gap*. We know that *First* $k$-*Gap* is efficient, *k*-stable and determined, therefore we know $\bigcap _{\mathcal {R}^* \in S(\mathcal {A})} \mathcal {R}^*(E) \subseteq \mathcal {R}(E)$.

Now, every determined voting rule must have a non-empty set of winners. If the voting rule is efficient, the set of winners must contain at least one top ranked alternative. Now, consider an enumeration of the alternatives $$c_1, \dots ,c_m$$ such that $$ sc _E(c_j) \ge sc _E(c_{j+1})$$ holds for all *j*. If a voting rule is *k*-stable, a winner set containing one top ranked alternative must contain all alternatives $$c_i$$ for which $$ sc _E(c_j) < sc _E(c_{j+1}) +k$$ holds for all $$j < i$$. By the definition of *First* $$k$$-*Gap* this implies $$\mathcal {R}(E) \subseteq \bigcap _{\mathcal {R}^* \in S(\mathcal {A})} \mathcal {R}^*(E)$$.

The minimality of *Approval Voting* is a special case of the minimality of *First* $k$-*Gap*, as 1-Stability equals Non-tiebreaking and *First-1-Gap* is equivalent to *Approval Voting*. $\square$

This result is another strong indication that *First* $k$-*Gap* is promising from an axiomatic standpoint. It produces shortlists that are as short as possible without violating *k*-Stability, an axiom that is desirable in many shortlisting scenarios.

Let us now consider larger sets of axioms. First, observe that for every set of axioms $\mathcal {A}$, adding any axiom that is already satisfied by the minimal voting rule for $\mathcal {A}$ does not change the minimal voting rule.

#### Proposition 7

Let $\mathcal {A}$ be a set of axioms, let $\mathcal {R}$ be the minimal voting rule for $\mathcal {A}$ and let *A* be an axiom that is satisfied by $\mathcal {R}$. Then $\mathcal {R}$ is the minimal voting rule for $\mathcal {A}\cup \{A\}$.

#### Proof

First, observe that $\mathcal {R}$ satisfies *A* and therefore $\mathcal {R}\in S(\mathcal {A} \cup \{A\})$. Moreover, by definition $S(\mathcal {A} \cup \{A\}) \subseteq S(\mathcal {A})$. It follows that for all elections *E* we have

$$\begin{aligned}\mathcal {R}(E) \supseteq \bigcap _{\mathcal {R}^* \in S(\mathcal {A} \cup \{A\})} \mathcal {R}^*(E) \supseteq \bigcap _{\mathcal {R}^* \in S(\mathcal {A})} \mathcal {R}^*(E)= \mathcal {R}(E).\end{aligned}$$

$$\square $$

As *Approval Voting* satisfies all axioms studied in this paper except $\ell$-Stability and Independence, it is the minimal determined shortlisting rule for each set of axioms not containing $\ell$-Stability or Independence. Moreover, *First* $k$-*Gap* satisfies every axiom except Anti-Unanimity and Independence. Therefore, it is the minimal $\ell$-stable shortlisting for every set of axioms not containing Anti-Unanimity or Independence. Now, we observe that Determined and Efficiency together clearly imply Unanimity. As we know from Theorem 3 that there is no shortlisting rule that satisfies $\ell$-Stability, Unanimity and Anti-Unanimity, there is also no minimal shortlisting rule that is determined and satisfies $\ell$-Stability and Anti-Unanimity.

Finally, the rule constantly returning the empty set is a shortlisting rule that satisfies independence, hence it is also the minimal rule satisfying our basic axioms and Independence. Additionally, it follows from Theorem 4 that the only determined rule that satisfies Independence is the rule that always returns the set of all candidates. Therefore, it is the unique rule in the set $S(\mathcal {A})$ where $\mathcal {A}$ consists of Anonymity, Neutrality, Non-tiebreaking, Efficiency, Determined and Independence and hence also the minimal rule for $\mathcal {A}$.

In summary, we can characterize the minimal voting rule for any subset of the axioms considered in this paper that at least contains our four basic axioms as well as Determined as follows:

#### Theorem 8

Let $\mathcal {A}$ be a subset of the axioms consider in this paper such that $\mathcal {A}$ contains Neutrality, Anonymity, Efficiency, Non-tiebreaking and Determined. Then the minimal voting rule for $\mathcal {A}$

- is *Approval Voting* if $\mathcal {A}$ contains neither Independence nor $\ell$-Stability.
- is *First* $k$-*Gap* if $\mathcal {A}$ contains $\ell$-Stability and neither Independence nor Anti-Unanimity.
- is the rule always returning all candidates if $\mathcal {A}$ contains Independence.
- does not exist if $\mathcal {A}$ contains both $\ell$-Stability and Anti-Unanimity.

## Experiments

In numerical experiments, we want to evaluate the characteristics of the considered shortlisting rules. The Python code used to run these experiments is available (Lackner and Maly 2022). We use three data sets for our experiments: two synthetic data sets (“bias model” and “noise model”) as well as data from a real-world shortlisting scenario, the nomination process for the Hugo awards.

### Synthetic data

#### Basic setup

Both synthetic data sets have the same basic setup. We assume a shortlisting scenario with 100 voters and 30 alternatives. Each alternative *c* has an objective quality $q_c$, which is a real number in [0, 1]. For each alternative, we generate $q_c$ from a truncated normal (Gauss) distribution with mean 0.75 and standard deviation 0.2, restricted to values in [0, 1]. This is chosen to model difficult shortlisting scenarios with several strong candidates (with an objective quality $q_c$ close to 1). Our base assumption is that voters approve an alternative with likelihood $q_c$. Thus, the approval score of alternatives are binomially distributed, specifically $sc _E(c)\sim B(100, q_c)$. We then modify this assumption to study two complications for shortlisting: imperfect quality estimates (noise) and biased voters.

#### The noise model

This model is controlled by a variable $\lambda \in [0,1]$. We assume that voters do not perfectly perceive the quality of alternatives, but with increasing $\lambda$ fail to differentiate between alternatives. Instead of our base assumption that each voter approves an alternative *c* with likelihood $q_c$, we change this likelihood to $(1-\lambda )q_c + 0.5\lambda$. Thus, for $\lambda =0$ this model coincides with our base assumption; for $\lambda =1$ we have complete noise, i.e., all alternatives are approved with likelihood 0.5. As $\lambda$ increases from 0 to 1, the amount of noise increases, or, in other words, the voters become less able to judge the quality of alternatives.

#### The bias model

In this model we assume that a proportion of the voters are biased against (roughly) half of the alternatives; we call these alternatives disadvantaged. Biased voters approve these alternatives only with likelihood $0.5\cdot q_c$, i.e., they perceive their quality as only half of their true quality. We assign each alternative with likelihood 0.5 to the set of disadvantaged alternatives. In addition, the alternative with the highest quality is always disadvantaged.^8^ We control the amount of bias via a variable $\gamma \in [0,1]$: a subset of voters of size $\left\lfloor 100\cdot \gamma \right\rfloor$ is biased; for the remaining voters our base assumption applies. As in the noise model, as $\gamma$ increases from 0 to 1 the shortlisting task becomes harder as the approval scores less and less reflects the actual quality of alternatives.

#### Instances

For each of the two models, we generate 1, 000 instances for each $\lambda \in \{0, 0.05, 0.1, \ldots , 0.95, 1\}$, thus resulting in 20,000 instances per model.

### The Hugo awards data set

The Hugo Awards are annual awards for works in science-fiction. Each year, awards are given in roughly 20 categories. The Hugo awards are particularly interesting for our paper as the nomination of candidates is based on voting and the submitted votes are made publicly available (this distinguishes the Hugo awards from many other literary awards with confidential nomination procedures).

**Fig. 1:**
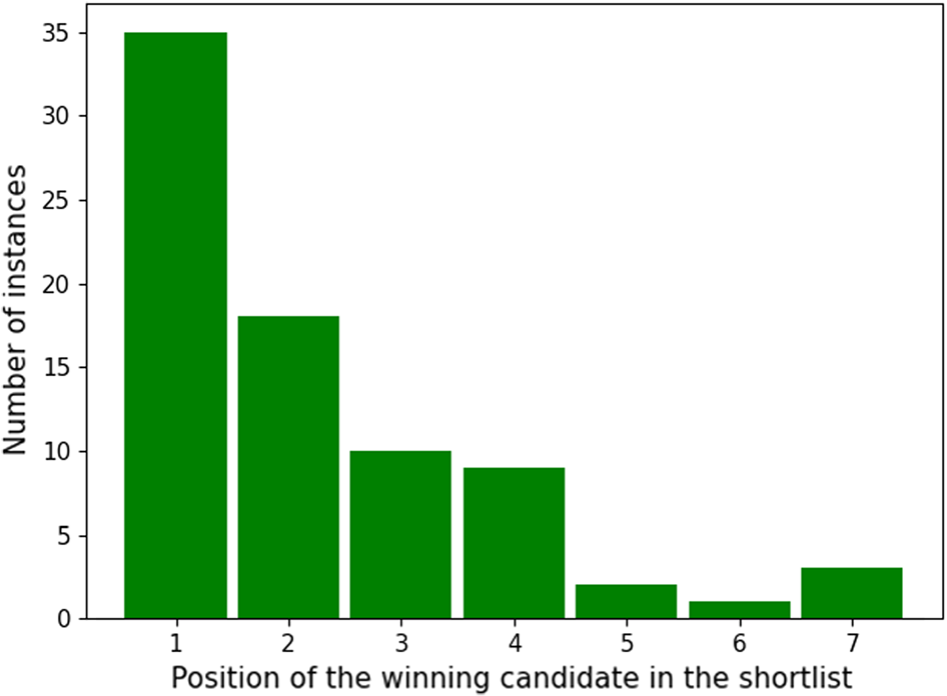
Shortlist positions of the actual winners when sorted by approval scores

The Hugo shortlisting (nomination) process works as follows. Each voter can nominate up to five candidates per category. This yields an approval-based election exactly as defined in Sect. 2. For each category, a shortlist of (usually) six candidates is selected. This shortlist, however, does not necessarily consist of the six candidates with the largest approval scores. Instead, a voting rule called “E Pluribus Hugo” is used. This is not a shortlisting rule in our sense (Definition 1), since it is not Non-tiebreaking and fails Efficiency.^9^ However, “E Pluribus Hugo” generally selects candidates with high approval scores and hence the actual winners are always among the top-seven candidates with the largest approval scores. In Fig. 1, we display in which position (when sorted by approval scores) the actual winner in the second stage is found. Note that there are three instances where a candidate in position 7 is winning. As “E Pluribus Hugo” always selects six candidates, this shows that either Non-tiebreaking or Efficiency is violated in these instances.

Our data set is based on the years 2018–2021, comprising a total of 78 shortlisting elections. The voting data for these years is publicly available on the Hugo website (https://www.thehugoawards.org/). For each election we recorded the actual winner in the second stage (also based on voting, but with a different, larger set of voters). The data files are available along-side our code (Lackner and Maly 2022).

In a sense this is an ideal data set to test our results, as the scenario exactly matches our formal model. However, there are two caveats to be noted. First, the true winner is always among the first seven candidates. Thus, *ISP-7* will always select a shortlist containing the true winner. Conversely, any shortlisting rule that outputs shortlists with more than seven candidates is non-optimal on this data set. This peculiarity has to be kept in mind when interpreting our results.

Secondly, the shortlisting process of the Hugo awards has been a contentious matter with recorded attempts of organized strategic voting [this is described briefly by Quinn and Schneier (2016)]. As a consequence, the voting results in the shortlisting stage can differ significantly from the results in the second stage (with a much larger set of voters). It is therefore reasonable to assume that this data set contains “hard” instances, i.e., it is difficult to find short shortlists.

### Precision and average size

We use two metrics to evaluate shortlisting rules. To be able to speak about successful shortlisting, we assume that we know for each shortlisting instance $E_\ell$ the actual winner in the second stage, i.e., the candidate that is the winner *among* shortlisted candidates; let this candidate be $c^*_\ell$. For the synthetic data sets, $c^*_\ell$ is the candidate with the highest objective quality; for the Hugo data set it is the candidate that actually won the Hugo award (which was selected from the shortlisted candidates).

Given a set of shortlisting instances $\{E_1,\dots ,E_N\}$, we evaluate a shortlisting rule $\mathcal {R}$ with respect to the following two metrics.

1. *Precision* is the true winner ($c^*_\ell$) being contained in $\mathcal {R}$’s winner sets:
  1 $$\begin{aligned} \frac{1}{N}\cdot \left|\left\{ 1\le \ell \le N : c^*_\ell \in \mathcal {R}(E_\ell ) \right\} \right|. \end{aligned}$$
2. *Average size* is the average size of $\mathcal {R}$’s winner sets:
  2 $$\begin{aligned} \frac{1}{N}\sum _{\ell =1}^N |\mathcal {R}(E_i)|. \end{aligned}$$

A shortlisting is desirable if it has a high precision and small average size. However, observe that these two metrics are difficult to reconcile. The easiest way to achieve high precision is to output large shortlists, and conversely, a small average size will likely result in a lower precision.^10^

### Experiment 1: increasing noise and bias

Experiment 1 applies only to the two synthetic data sets. The goal is to see how different shortlisting rules deal with increasingly noisy/biased data. We restrict our attention to six shortlisting rules, for which the results are particularly instructive: *Approval Voting*, *First* $5$-*Gap*, $f$-*Threshold*, *Size Priority*, *First Majority*, *Top-10-First-5-Gap*, *Largest Gap*, and 0.5-*NCSA*. For *Size Priority*, we use the priority order $4\rhd 5 \rhd 6 \rhd \dots$, i.e., we use the *ISP-4* rule. Finally, we choose $0.5n$-*Threshold* as representative for threshold rules. The results for *Max-Score-f-Threshold* with $f(x)=0.5x$ were very similar to $0.5n$-*Threshold* and are thus omitted.

**Fig. 2:**
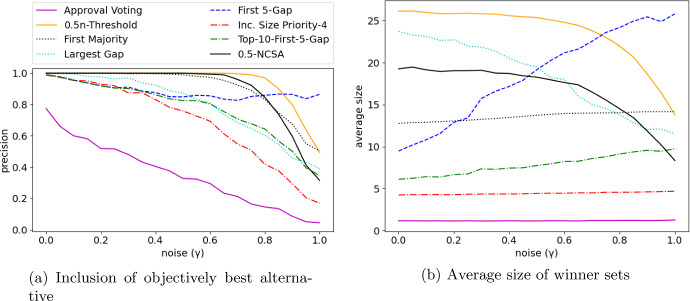
Numerical simulations for the noise model

**Fig. 3:**
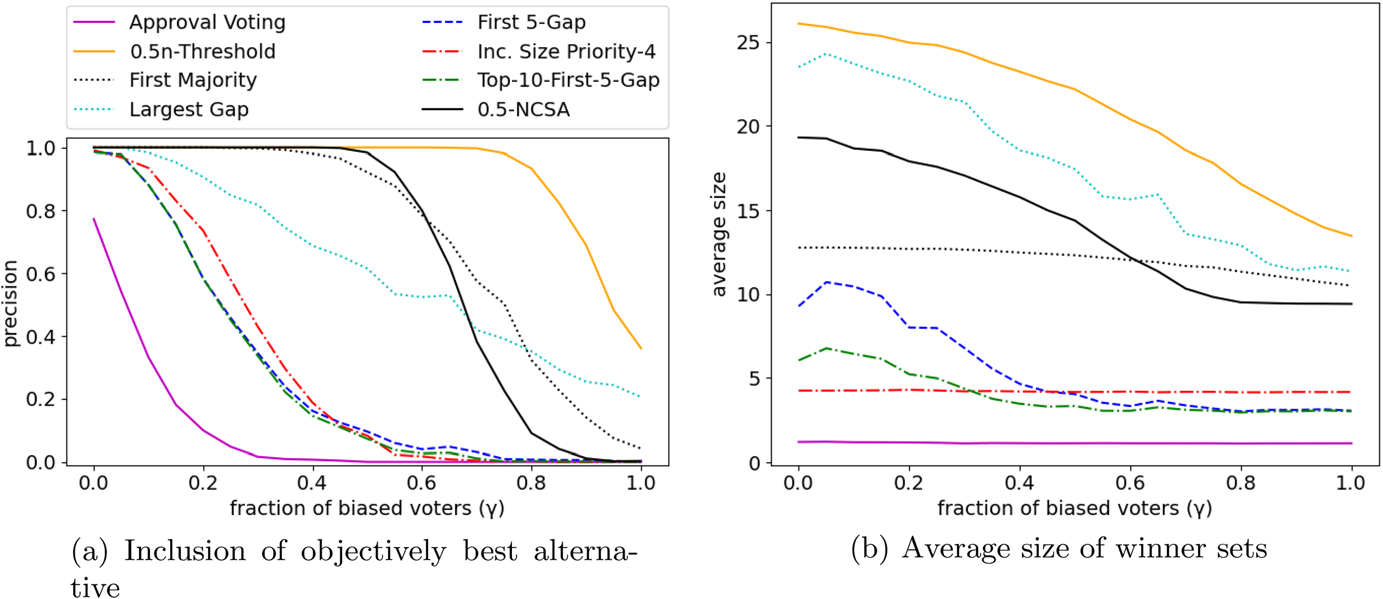
Numerical simulations for the bias model

Our comparison of shortlisting rules is visualized in Fig. 2 for the noise model and Fig. 3 for the bias model. Each data point in these figures (corresponding to a specific $\lambda$) is based on $N=1000$ instances $E_1,\dots , E_{N}$.

The orthogonal nature of precision and average seen can be seen clearly when comparing *Approval Voting* and $0.5n$-*Threshold*: *Approval Voting* returns rather small winner sets (as seen in Figs. 2b and 3b), but if $\lambda$ increases, the objectively best alternative is often not contained in the winner set (Figs. 2a and 3a). $0.5n$-*Threshold* has large winner sets, but is likely to contain the objectively best alternative even for large $\lambda$ (up to $\lambda \approx 0.8$). If the average size of winner sets remains roughly constant (*Increasing Size Priority*, *First Majority*, *Approval Voting*), then the precision reduces with increasing noise/bias ($\lambda$).

*Size Priority* (with the considered priority order) is a noteworthy alternative to *Approval Voting*. It has an only slightly larger average size (roughly 1 vs 4), while having a significantly larger chance to include the objectively best alternative. As it is generally not necessary to have extremely small winner sets in shortlisting processes, we view *Size Priority* (with a sensibly chosen priority order) as superior to *Approval Voting*.

Considering the noise model (Fig. 2), we see a very interesting property of *First* $5$-*Gap*: it is the only rule where the size of winner sets significantly adjusts to increasing noise. If $\lambda$ increases, the differences between the approval scores vanishes and thus fewer 5-gaps exist. As a consequence, the winner sets increase in size. This is a highly desirable behavior, as it allows *First* $5$-*Gap* to maintain a high likelihood of containing the objectively best alternative without producing very large shortlists for low-noise instances.

Two other rules also show this behavior: *Top-10-First-5-Gap* and *First Majority*, albeit both to only a small degree. *Top-10-First-5-Gap* achieves the same precision as *First* $5$-*Gap* until $\gamma$ reaches $\approx 0.5$ after which its precision deteriorates. On the other hand, note that *Top-10-First-5-Gap* has a considerably smaller average size. For *Largest Gap*, $0.5n$-*Threshold*, and *q-NCSA*, we see the opposite effect: winner sets are large for low noise but decrease with increasing $\lambda$. This is not a sensible behavior; note that *First Majority* achieves better precision with much smaller average size.

For the bias model, we do not observe any shortlisting rule that reacts to an increase in bias with a larger average size.

To sum up, our experiments show the behavior of shortlisting rules with accurate and inaccurate voters, and the tradeoff between large and small winner set sizes. In these experiments, we see two shortlisting rules with particularly favorable characteristics:

1. *Size Priority* produces small winner sets with good precision. Thus, it shows a certain robustness to a noisy selection process, as is desirable in shortlisting settings.
2. *First* $k$-*Gap* manages to adapt in high-noise settings by increasing the winner set size, the only rule with this distinct feature. This makes it particularly recommendable in settings with unclear outcomes (few or many best alternatives), where a flexible shortlisting method is required. As we will see in the next experiment, however, *First* $k$-*Gap* on its own can be insufficient, which leads us to recommending the related *Top*-$s$-*First*-$k$-*Gap* rule instead.

### Experiment 2: tradeoffs between precision and size

**Fig. 4:**
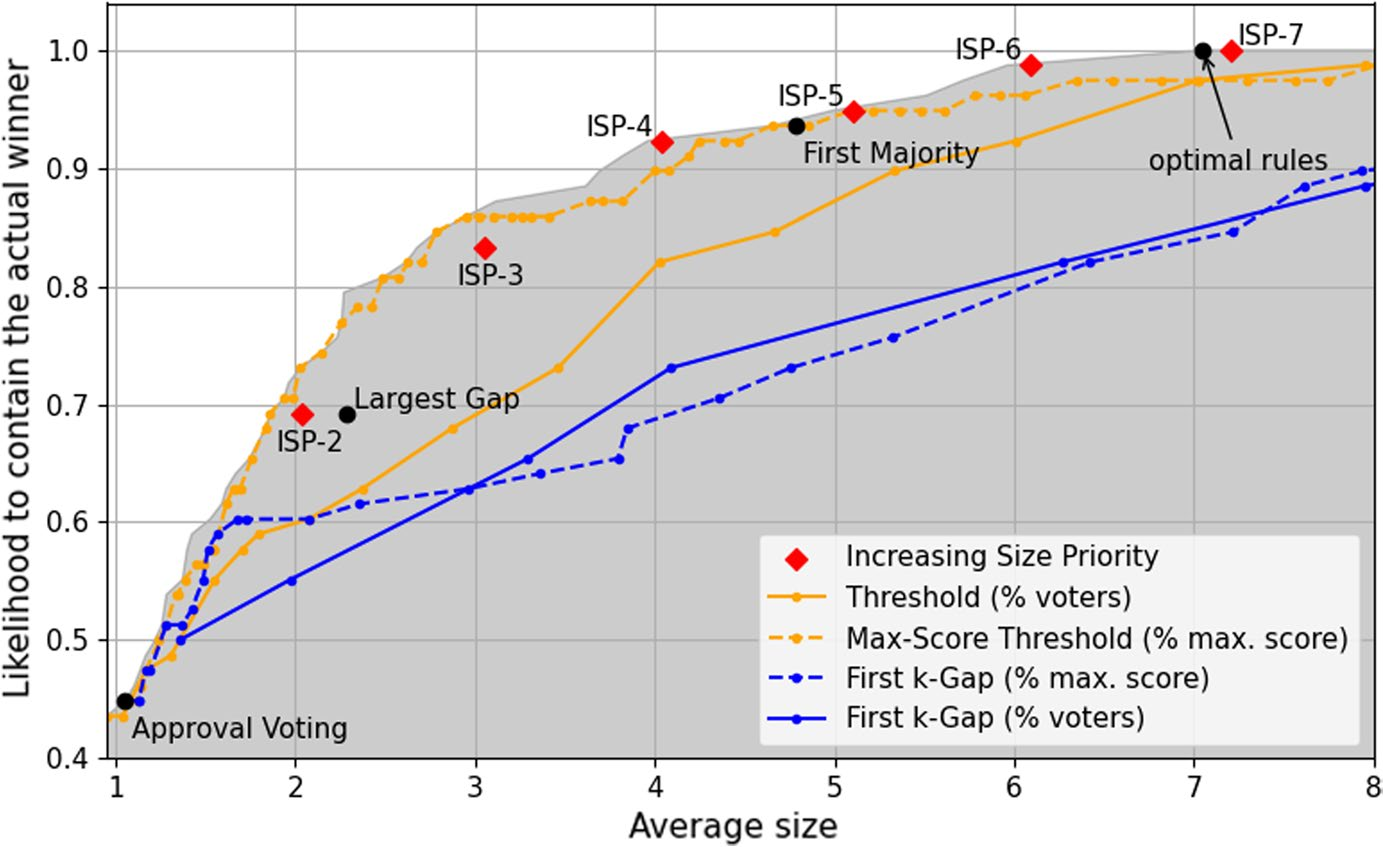
Results for the Hugo data set (Experiment 2)

**Fig. 5:**
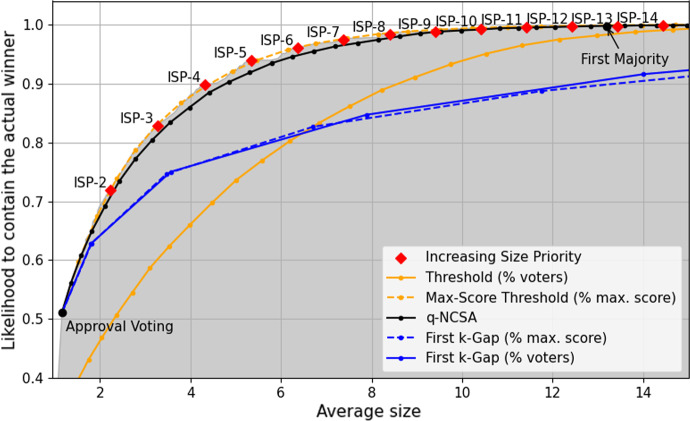
Results for the noise model (Experiment 2)

**Fig. 6:**
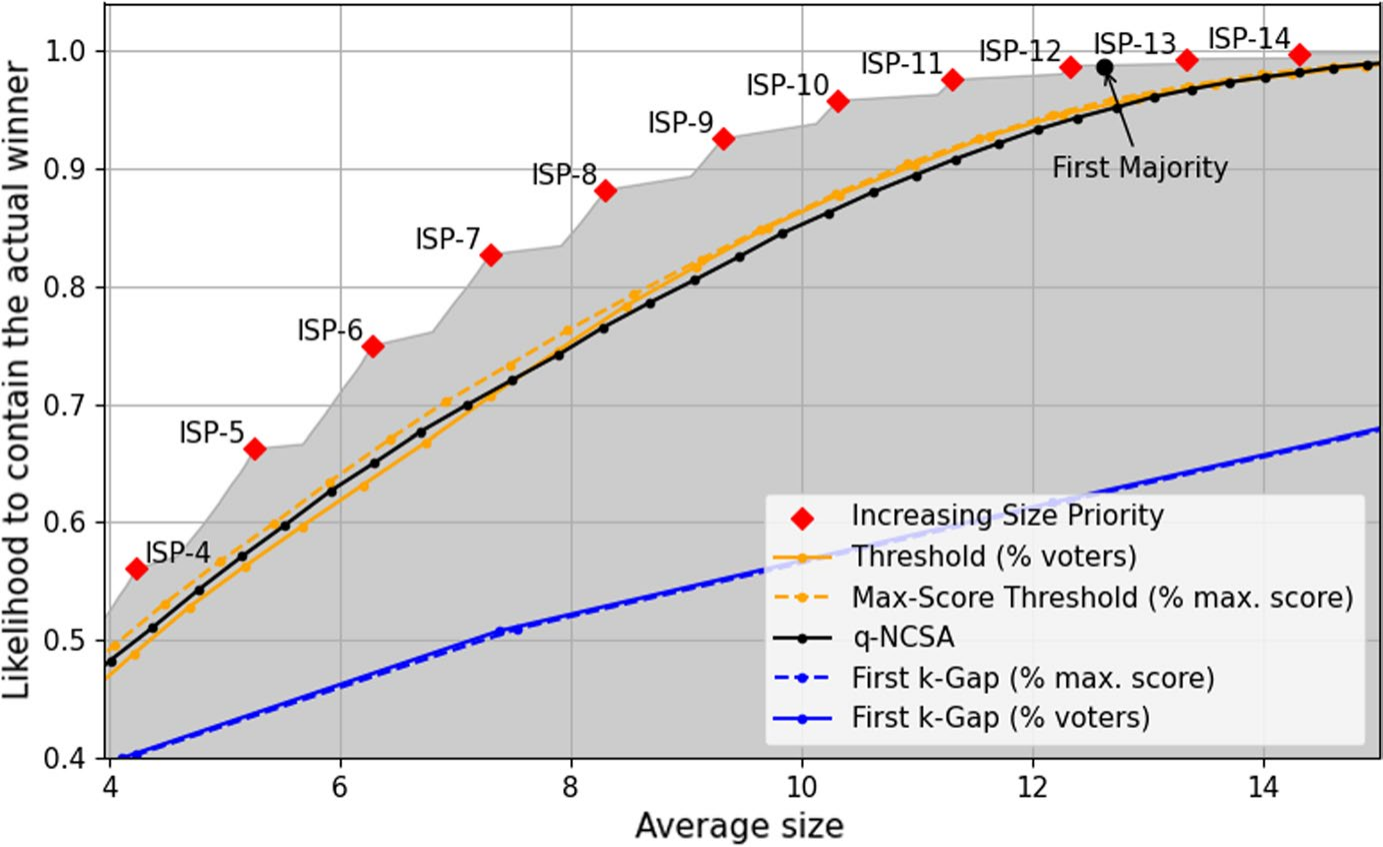
Results for the bias model (Experiment 2)

In this second experiment, we want to study the tradeoff between precision and size in more depth and for many more shortlisting rules. Here, we put particular emphasis on the Hugo data set (but also consider both synthetic sets). To this end, we represent shortlisting rules as points in a two-dimensional plane with average size as *x*-axis and precision as *y*-axis. Figure 4 shows these results for the Hugo data set (points are averaged over 78 instances), Fig. 5 shows these results for the noise data set (no noise to moderate noise, i.e., $\lambda \in [0,0.5]$, yielding 10,000 instances), and Fig. 6 for the bias model (also for $\lambda \in [0,0.5]$, 10,000 instances).

These plots can be understood as follows. Ideal shortlisting rules lie in the top left corner (high precision, low average size). As this is generally unachievable, we have to choose a compromise between the two metrics. The gray area shows the space in which such a compromise has to be found (when choosing from shortlisting rules that are studied in this paper).

We will now explain the gray area in more detail: For Experiment 2, we consider all shortlisting rules defined in Sect. 3 with the following parameters. For $\alpha \in \{0,0.01, 0.02, \dots , 1\}$, we consider

- *Next*-$$k$$ for $$k\in \{2,3\}$$,
- $f$-*Threshold* and *Max-Score-f-Threshold* with $f(n)=\lfloor \alpha \cdot n\rfloor$,
- *q-NCSA* with $$q=\alpha $$,
- *First* $k$-*Gap* with $k=\lfloor \alpha \cdot n \rfloor$ and with $k=\lfloor \alpha \cdot \max { sc (E)}\rfloor$,
- *Increasing Size Priority* with priority orders of the form $s\rhd s+1\rhd \dots$ (*ISP-s*) for $2\le s\le m$,
- *Top*-$s$-*First*-$k$-*Gap* with $2\le s\le m$ and $k \in \{\lfloor \alpha \cdot n \rfloor , \lfloor \alpha \cdot \max { sc (E)}\rfloor \}$.

Each shortlisting rule yields a point in this two-dimensional space. Shortlisting rules with one parameter are displayed as lines. We can compute a Pareto frontier consisting of all points that do not have another point above and to the left of it. The boundary of the gray area shows this Pareto frontier. Consequently, voting rules close to this frontier represent a more beneficial tradeoff between precision and average size.

#### Results for the Hugo data set

When looking at Fig. 4, we see as expected that *ISP-7* achieves a precision of 1 and an average size slightly above 7 (due to ties). We furthermore see that *ISP-4*, *ISP-5*, and *ISP-6* are all very close to the Pareto frontier. This raises the question whether *Increasing Size Priority* is an ideal choice for this data set. While this class is a good choice, it can be improved by *Top*-$s$-*First*-$k$-*Gap*. In Table 3, we exemplarily show the precision and average size values for *ISP-6*, and *ISP-7* alongside shortlisting rules that achieve a smaller average size with the same (or better) precision. This table gives an indication how to use *Top*-$s$-*First*-$k$-*Gap*in a real-world shortlisting task: First, choose a sensible maximum size of a shortlist; in the case of Hugo awards this was chosen to be six (and was five prior to 2017). Then, identify a bound that constitutes a significant gap; this bound can be chosen conservatively. In the Hugo data set, a sensible choice appears to be $30\%$ of voters. That is, if we encounter a gap (in the sense of *First* $k$-*Gap*) in $(E)$ of more than 0.3*n*, we cut the shortlist at this point if this leads to a shorter shortlist.

**Table 3:** Shortlisting rules that are superior to *ISP-6* and *ISP-7* in the Hugo data set

Shortlisting rule	Average size	Precision
*ISP-7*	7.205	1.000
*Top*-$7$-*First*-$\lfloor \alpha \cdot n \rfloor$-*Gap* for $\alpha \in [0.31,0.39]$	7.128	1.000
*Top*-$7$-*First*-$\lfloor \alpha \cdot \max { sc (E)}\rfloor$-*Gap* for $\alpha \in [0.70,0.72]$	7.128	1.000
*Top*-$7$-*First*-$\lfloor \alpha \cdot n \rfloor$-*Gap* for $\alpha \in [0.27,0.30]$	7.051	1.000
*Top*-$7$-*First*-$\lfloor \alpha \cdot \max { sc (E)}\rfloor$-*Gap* for $\alpha =0.69$	7.051	1.000
*ISP-6*	6.090	0.987
*Top*-$6$-*First*-$\lfloor \alpha \cdot n \rfloor$-*Gap* for $\alpha \in [0.31,0.39]$	6.026	0.987
*Top*-$6$-*First*-$\lfloor \alpha \cdot \max { sc (E)}\rfloor$-*Gap* for $\alpha \in [0.70,0.72]$	6.026	0.987
*Top*-$6$-*First*-$\lfloor \alpha \cdot n \rfloor$-*Gap* for $\alpha \in [0.27,0.30]$	5.962	0.987
*Top*-$6$-*First*-$\lfloor \alpha \cdot \max { sc (E)}\rfloor$-*Gap* for $\alpha =0.69$	5.962	0.987

Let us now consider other shortlisting rules. We see that *Max-Score*-$f$-*Threshold* closely traces the Pareto frontier and thus is a very good choice for selecting a compromise between precision and average size. $f$-*Threshold* and *First* $k$-*Gap* are less convincing. *q-NCSA* performs even worse, as very often candidates have approval scores of less than 0.5*n*. Therefore *q-NCSA* selects mostly empty sets and is thus not visible in Fig. 4 (cf. Observation 2). A notable unparameterized rule is *First Majority*, which is very close to the Pareto frontier.

To sum up our results for the Hugo data set, we identify the following shortlisting rules as particularly suitable. *Top*-$7$-*First*-$\lfloor \alpha \cdot n \rfloor$-*Gap* for $\alpha \in [0.27,0.30]$ and *Top*-$7$-*First*-$\lfloor 0.69 \cdot \max { sc (E)}\rfloor$-*Gap* achieve a precision of 1 with the smallest average size (7.051); in Fig. 4 these rules correspond to the point labeled “optimal rules”. In general, *Increasing Size Priority* and *Max-Score*-$f$-*Threshold* achieve a very good compromises between precision and average size.

#### Results for the noise and bias models

Figure 5 shows the results for the noise model. We see that also here *Increasing Size Priority* and *Max-Score*-$f$-*Threshold* are very close to the Pareto frontier. The same holds for *First Majority*. A major difference to the Hugo data set is the performance of *q-NCSA*. As candidates generally have approval scores of more than 0.5*n*, *q-NCSA*works as intended with points close to the Pareto frontier. As before, $f$-*Threshold* and *First* $k$-*Gap* are less convincing.

The bias model is a scenario, where some high-quality candidates receive too few approvals. In Fig. 6, we see that this is a tough problem. The only recommendable shortlisting rules are *Increasing Size Priority* rules. By simply shortlisting the top-*k* candidates, there is a certain chance to also shortlist high-quality but disadvantaged candidates. We remark that the Pareto frontier between *ISP–*points is due to *Top*-$s$-*First*-$k$-*Gap* rules.

## Discussion

We conclude this paper by condensing our analysis to obtain recommendations for shortlisting methods. First, however, we briefly discuss a connection between clustering algorithms and shortlisting.

### Clustering algorithms as shortlisting methods

One can view the goal of shortlisting as classifying some alternatives as most suitable. The machine learning literature offers a wide variety of clustering algorithms that can perform such a classification. In the following, we describe how clustering algorithms can be translated into an approval-based variable multi-winner rule that satisfies Anonymity. For most clustering algorithms, the corresponding rule also satisfies Neutrality, Efficiency and is non-tiebreaking, and thus yields a shortlisting method. The procedure works as follows: We use $$ sc (E)$$ as input for a clustering algorithm (but these scores could also be derived from sources other than approval ballots). The clustering algorithm produces a partition $$S_1,\dots ,S_\beta $$ of $$ sc (E)$$. We define the winner set as the set $$S \in \{S_1,\dots ,S_\beta \}$$ that contains the highest score, or, to be more precise, the winner set consists of those candidates whose scores are contained in *S*.

As this procedure is based on $$ sc (E)$$, the resulting approval-based variable multi-winner rule is clearly anonymous. To show that the resulting rule is a shortlisting rule, we require the following two additional assumptions:

1. *The clustering algorithm yields the same result for any permutation of* $$ sc (E)$$. If this is the case, the resulting rule is also neutral.
2. *The algorithm outputs clusters that are non-intersecting intervals.* If this is the case, the result rule is non-tiebreaking (since clusters do not intersect). It is also efficient, as the “winning” cluster is an interval containing the largest score.

These are indeed conditions that any reasonable clustering algorithm satisfies.

As an illustration, let us consider linkage-based algorithms (Shalev-Shwartz and Ben-David 2014). Linkage-based algorithms work in rounds and start with the partition of $$ sc (E)$$ into singletons. Then, in each round, two sets (clusters) are merged until a stopping criterion is satisfied. One important type of linkage-based algorithms are those where always the two clusters with minimum distance are merged. Thus, such algorithms are specified by two features: a distance metric for sets (to select the next sets to be merged) and a stopping criterion. We assume that if two or more pairs of sets have the same distance, then the pair containing the smallest element are merged. Following Shalev-Shwartz and Ben-David (2014), we consider three distance measures: the minimum distance between sets (Single Linkage):

3 $$\begin{aligned} d_{\min }(A,B)&= \min \left\{ |x-y|: x\in A, y\in B\right\} , \end{aligned}$$

the average distance between sets (Average Linkage)

4 $$\begin{aligned} d_{\textrm{aver}}(A,B)&= \frac{1}{|A||B|}\sum _{x\in A, y\in B} |x-y|, \end{aligned}$$

and the maximum distance between sets (Max Linkage)

5 $$\begin{aligned} d_{\max }(A,B)&= \max \left\{ |x-y|: x\in A, y\in B\right\} . \end{aligned}$$

These three methods can be combined with arbitrary stopping criteria; we consider two: (A) stopping as soon as only $\beta$ clusters remain, and (B) stopping as soon as every pair of clusters has a distance of $\ge \alpha$. Interestingly, two of our previously proposed methods correspond to linkage-based algorithms: First, if we combine the minimum distance with stopping criterion (A) for $\beta =2$, we obtain the *Largest Gap* rule. Secondly, if we use the minimum distance and impose a distance upper-bound of $\alpha =k$ (stopping criterion B), we obtain the *First* $k$-*Gap* rule. Another interesting method resulting from this approach is combining stopping criterion (A) with $\beta =k$: this method results in winner sets of size roughly *m*/*k* and is thus related to *Size Priority*.

We see that the literature on clustering algorithms yields a large number of shortlisting methods. The inherent disadvantage of this approach is that cluster algorithms generally treat all clusters as equally important whereas for shortlisting methods the winning set of candidates is clearly most important. This difference becomes most pronounced when a clustering algorithm produces several clusters; only the “winning” cluster is relevant for the resulting shortlisting method, all further clusters are irrelevant for shortlisting.

On the other hand, clustering algorithms are more flexible and are suitable for more complex inputs than $$ sc (E)$$, e.g., more-dimensional input. If the quality of candidates is measured by several numeric scales (possibly only one of them related to voting), the flexibility of clustering algorithms becomes relevant. In addition, the work on *fair clustering* (Chhabra et al. 2021) can be relevant for shortlisting. Fairness in this context generally refers to additional constraints on valid clusterings. An example could be the requirement that a book shortlist should contain an equal proportion of fiction and non-fiction. Note that such constraints are concerned with fairness for candidates and not voters. Also, such constraints are in conflict with our Efficiency axiom (Sect. 2); this axiom would have to be weakened substantially to make it compatible with this kind of fairness.^11^

### Recommendations

The choice of a shortlisting rule has to be made in consideration of the application scenario. Each application comes with its own desiderata, which can be compared to the axiomatic results obtained here (Table 2). Moreover, there might be practical restrictions on the complexity of voting rule, i.e., how difficult it is to understand the voting process. We view most of the rules considered as suitable for practical use (with the possible exception of *q-NCSA*). Note that the explanation of a voting rule does not necessarily require a detailed rationale for the choice of parameter values. That is, parameterized rules (*First* $k$-*Gap*, *Top*-$s$-*First*-$k$-*Gap*, *Size Priority*, etc.) might be difficult to explain as classes of voting rules, but are much simpler when instantiated with concrete values.

Overall, we recommend three shortlisting methods based on our analysis: *Size Priority*, *Top*-$s$-*First*-$k$-*Gap*, and $f$-*Threshold*. Let us discuss their advantages and disadvantages:

- *Size Priority*, in particular *Increasing Size Priority*, is recommendable if the size of the winner set is of particular importance, e.g., in highly structured shortlisting processes such as the nomination for awards. *Increasing Size Priority* exhibits good axiomatic properties (cf. Table 2) as well as a very solid behavior in our numerical experiments. In particular for the bias data set, where a (unknown) subset of candidates is discriminated against, *Increasing Size Priority* appears to be the best choice. By selecting *k* candidates with the highest approval scores (or more in case of ties), the differences in approval scores within the selected group are ignored and thus disadvantaged, high-quality candidates have a better chance to be chosen. On the other hand, *Increasing Size Priority* makes limited use of the available approval preferences and thus can be seen as a good choice mostly in settings with limited trust in voters’ accuracy. When voters are expected to have good estimates of the candidates’ qualities, the following two shortlisting rules are better suited.
- Our axiomatic analysis reveals *First* $k$-*Gap* as a particularly strong rule in that it is the minimal rule satisfying $\ell$-Stability. Furthermore, it is the only rule that adapts to increasing noise in our simulations. However, we have seen in Experiment 2 (Sect. 5.5) that *First* $k$-*Gap* is prone to choosing winner sets that are larger than necessary. Thus, we recommend to use *Top*-$s$-*First*-$k$-*Gap* instead. *Top*-$s$-*First*-$k$-*Gap* shares most axiomatic properties with *First* $k$-*Gap* (cf. Table 2) except $\ell$-Stability and Resistance to Clones. Another advantage of *Top*-$s$-*First*-$k$-*Gap* is that the parameter *k* is difficult to choose for *First* $k$-*Gap*, whereas it is very reasonable to conservatively pick a large *k*-value for *Top*-$s$-*First*-$k$-*Gap*. Choosing *k* too large simply diminishes the differences between *Top*-$s$-*First*-$k$-*Gap* and *ISP-k*.
- Finally, Theorem 4 shows that $f$-*Threshold* rules are the only rules satisfying the Independence axiom. Therefore, if the selection of alternatives should be independent from each other, then clearly a $f$-*Threshold* rule should be chosen. For example, the inclusion in the Baseball Hall of Fame should depend on the quality of a player and not on the quality of the other candidates. In our experiments, we have seen that the related class of *Max-Score*-$f$-*Threshold* rules has advantages over $f$-*Threshold* rules. The difference between these two classes, however, is only relevant if the maximum score of candidates differs between elections for reasons unrelated to the candidates’ quality. This was the case, e.g., in the Hugo data set, where the relative maximum approval score varied significantly between award categories.

### Future work

Our recommendations are applicable to most shortlisting scenarios. There are, however, possible variations of our shortlisting framework that require further analysis. For example, while strategyproofness is usually not important in election with independent experts, there are some shortlisting applications with a more open electorate where this may become an issue (Quinn and Schneier 2016; Bredereck et al. 2017). We have not considered strategic voting in this paper and assume that this viewpoint will give rise to different recommendations. Moreover, it may be worth investigating whether using ordinal preferences (rankings) instead of approval ballots can increase the quality of the shortlisting process [shortlisting rules for ordinal preferences can be found, e.g., in the works of Elkind et al. (2017a, 2017b), Aziz et al. (2017b), Faliszewski et al. (2017)]. In general, the class of variable multi-winner rules (and social dichotomy functions) deserves further attention as many fundamental questions (concerning proportionality, axiomatic classifications, algorithms, etc.) are still unexplored.

## Supplementary Information

Below is the link to the electronic supplementary material.

Supplementary file 1 (png 51 KB)

Supplementary file 2 (png 46 KB)

Supplementary file 3 (png 60 KB)

Supplementary file 4 (png 51 KB)

Supplementary file 5 (png 47 KB)

Supplementary file 6 (png 50 KB)

Supplementary file 7 (png 43 KB)

Supplementary file 8 (png 76 KB)

Supplementary file 9 (png 71 KB)

Supplementary file 10 (png 76 KB)

Supplementary file 11 (png 70 KB)

Supplementary file 12 (png 178 KB)

Supplementary file 13 (png 174 KB)

Supplementary file 14 (png 176 KB)

Supplementary file 15 (png 171 KB)

Supplementary file 16 (png 44 KB)

## Data Availability

All data and code used in the paper is available here: 10.5281/zenodo.3821983.

## Appendix

### A. Original definition of *q-NCSA*

Faliszewski et al. (2020) defined the *q-NCSA*-score of a set of candidates $S\subseteq C$ for a real number $q \in [0,1]$ as follows:

$$\begin{aligned} sc _E^{\textit{q-NCSA}}(S)=\sum _{v \in V}\left( \frac{|S \cap A_v|}{|S|^q} - \frac{|S \cap \overline{A_v}|}{|S|^q}\right) . \end{aligned}$$

This is equivalent to the definition given in this work as the following computation shows, where $\mathbb {1}$ is the indicator function and $A_v$ the set of candidates approved by *v*:

$$\begin{aligned} \sum _{v \in V}\left( \frac{|S \cap A_v|}{|S|^q} - \frac{|S \cap \overline{A_v}|}{|S|^q}\right)&= \frac{1}{|S|^q} \sum _{v \in V}\left( \sum _{c\in S}\mathbb {1}_{c\in v} - \sum _{c\in S}(1-\mathbb {1}_{c\in v})\right) \\&\quad =\frac{1}{|S|^q}\sum _{c \in S} \left( \sum _{v\in V}\mathbb {1}_{c\in v} - \sum _{v\in V}(1-\mathbb {1}_{c\in v})\right) \\&\quad = \frac{1}{|S|^q}\sum _{c \in S}( sc _E(c) - (n - sc _E(c))) \\&\quad =\frac{1}{|S|^q}\sum _{c \in S}(2 sc _E(c) - n). \end{aligned}$$

### B. Analysis of axiom satisfaction

#### Unanimity, anti-unanimity, $\ell$-stability

Let us analyze the considered shortlisting rules with regard to the three axioms Unanimity, Anti-Unanimity and $\ell$-Stability.

- It is straightforward to see that *Approval Voting*, $f$-*Threshold*, *Max-Score*-$f$-*Threshold* and *Largest Gap* satisfy Unanimity and Anti-Unanimity for all non-degenerate profiles. Hence, they cannot satisfy $\ell$-Stability for $\ell > 1$.
- By definition, *First* $k$-*Gap* satisfies Unanimity and $\ell$-Stability for $k\ge \ell$ for all elections. Therefore, it cannot satisfy Anti-Unanimity.
- *First Majority* satisfies Unanimity as $$c_1 \in \mathcal {R}(E)$$ by definition and $$ sc _E(c_i) = \max _{c\in C}( sc _E(c)) = sc _E(c_1)$$ implies $$c_i \in \mathcal {R}(E)$$. Furthermore, we claim that it satisfies Anti-Unanimity. Let $$c_1, \dots , c_m$$ be the enumeration of the candidates used by *First Majority* and let $$c_i$$ be the first candidate with $$ sc _E(c_i) =0$$. Then, $$\sum _{j>(i-1)} sc _E(c_{j}) > 0$$ while $$\sum _{j >(i-1)} sc _E(c_{j}) = 0$$. This implies that $$c_i \not \in \mathcal {R}(E)$$. It follows that *First Majority* does not satisfy $$\ell $$-Stability for $$\ell >1$$.
- *Next*-$$k$$ satisfies Unanimity by definition. Furthermore, we claim that it satisfies Anti-Unanimity. Let $$c_1, \dots , c_m$$ be the enumeration of the candidates used by *Next*-$$k$$ and let $$c_i$$ be the first candidate with $$ sc _E(c_i) =0$$. Then, $$ sc _E(c_{i-1}) > 0$$ while $$\sum _{j = 1}^{k} sc _E(c_{(i-1)+j}) = 0$$. This implies that $$c_i \not \in \mathcal {R}(E)$$. As before, it follows that *Next*-$$k$$ does not satisfy $$\ell $$-Stability for $$\ell >1$$.
- *q-NCSA* satisfies Unanimity and Anti-Unanimity. First, we show that *q-NCSA* satisfies Unanimity: Let $$c_i$$ be a candidate with $$ sc _E(c_i) = n$$. Then, $$ sc _E^{\textit{q-NCSA}}(\{c_i\}) > 0 = sc _E^{\textit{q-NCSA}}(\emptyset )$$ and therefore $$\mathcal {R}(E) \ne \emptyset $$. As $$ sc _E(c_i) = \max _{c\in C}( sc _E(c))$$ this implies by Efficiency and Non-tiebreaking that $$c_i \in \mathcal {R}(E)$$. Now, we show that *q-NCSA* satisfies Anti-Unanimity: Let $$c_i$$ be a candidate with $$ sc _E(c_i) = 0$$. Then $$2 sc _E(c_i) -n < 0$$, which means for every set *S* such that $$c_i \in S$$ that the *q-NCSA*-score of *S* is strictly smaller than the *q-NCSA*-score of $$S \setminus \{c_i\}$$. This means that the *q-NCSA*-score of *S* is not maximal. As *S* was chosen arbitrarily, we can conclude $$c_i \not \in \mathcal {R}(E)$$. As above, it follows that *Next*-$$k$$ does not satisfy $$\ell $$-Stability for $$\ell >1$$.
- Now, we claim that *Size Priority* always satisfies either Unanimity or Anti-Unanimity: First, we show that it satisfies Unanimity if $$m \vartriangleright 0$$: As selecting all *m* candidates can never be tiebreaking, *Size Priority* will never select the empty set in this case. This implies $$c_1 \in \mathcal {R}(E)$$ for all *E*. As *Size Priority* is non-tiebreaking it follows that all $$c_i$$ with $$ sc _E(c_i)= n$$ must be in the winning shortlist. On the other hand, *Size Priority* satisfies Anti-Unanimity if $$0 \vartriangleright m$$ holds by a symmetric argument. Moreover, we claim that it satisfies both axioms (for non-degenerate profiles) if and only if it is a *Decisive Size Priority* rule. First let $$\mathcal {R}$$ be a *Decisive Size Priority* rule. Then, for every non-degenerate profile there must be a *i* such that $$c_1, \dots , c_i$$ can be selected as winners without tiebreaking. As $$\mathcal {R}$$ is a *Decisive Size Priority* rule, we know $$i \vartriangleright m$$ and $$i \vartriangleright 0$$. It follows that $$\mathcal {R}(E)$$ is neither *C* nor $$\emptyset $$. By a similar argument as before, this implies that $$\mathcal {R}$$ satisfies Unanimity and Anti-Unanimity. Now assume that $$\mathcal {R}$$ is a *Size Priority* instance that is not determined. Then there exists a $$0<k <m$$ such that either $$m \vartriangleright k$$ or $$0 \vartriangleright k$$. Consider an election *E* such that $$ sc _E(c_i) = n$$ for all $$i \le k$$ and $$ sc _E(c_j) =0$$ if $$j > k$$. Then, in the first case all candidates are winners and hence Anti-Unanimity is violated and in the second case no candidate is a winner and hence Unanimity is violated. It follows from the above that *Increasing Size Priority* satisfies Unanimity but not Anti-Unanimity. Finally, *Size Priority*, by definition, satisfies $$\ell $$-Stability for $$\ell > 1$$ if and only if 0 or *m* is the most preferred size.
- Finally, *Top*-$$s$$-*First*-$$k$$-*Gap* satisfies Unanimity because *First* $$k$$-*Gap* and *Increasing Size Priority* do so. That means for all election both winner sets $$W'$$ and $$W''$$ considered by *Top*-$$s$$-*First*-$$k$$-*Gap* satisfy unanimity. Hence, whatever set is chosen, unanimity is satisfied. On the other hand, it satisfies neither $$\ell $$-Stability for $$\ell > 1$$ nor Anti-Unanimity. Consider first the election given by $$ sc (E) = (3,2,0,0)$$ and *Top*-$$1$$-*First*-$$2$$-*Gap*. Then $$\{c_1\}$$ is the winner set and hence 2-Stability is violated. Now consider the same election under *Top*-$$3$$-*First*-$$3$$-*Gap*. Then both $$W'$$ and $$W''$$ equal $$\{c_1, \dots ,c_4\}$$ and hence Anti-Unanimity is violated.

#### Determined

As mentioned in the main text, all shortlisting rules considered in this paper, expect $f$-*Threshold*, *Size Priority* and *q-NCSA* are determined by definition.

- For $f$-*Threshold* it is clear that $\mathcal {R}(E)$ can be empty if no candidate achieves enough approvals to clear the threshold. Observe that this is not the case for *Max-Score*-$f$-*Threshold*, as we assume $f(n) < n$ and hence candidates with maximal score are always winners.
- *Size Priority* returns the empty set if 0 is the most preferred set size that does not require tiebreaking. This cannot happen if *Size Priority* is either a *Decisive Size Priority* rule or $m \vartriangleright 0$; *Size Priority* is determined in these cases. In particular this means that *Increasing Size Priority* is determined.
- For *q-NCSA* we observe that if no candidate has at least $50\%$ approvals then $2 sc _E(c) - n$ is negative for all candidates and hence the *q-NCSA*-score is only maximized by the empty set. Hence *q-NCSA* is not determined.

#### Independence of losing alternatives

Clearly, $f$-*Threshold* satisfies Independence of Losing Alternatives as it also satisfies Independence. As removing a losing alternative does not change the maximal score, the same holds for *Max-Score*-$f$-*Threshold*. Furthermore, as the removal of a losing alternative can only widen the gap between the winners and the non-winners, *First* $k$-*Gap* satisfies Independence of Losing Alternatives, and so does *Approval Voting*, which is a special case of *First* $k$-*Gap*. Finally, for *q-NCSA* the removal of a losing alternative just removes some non-maximal sets from consideration. Clearly, this does not change which sets have maximal *q-NCSA*-score.

None of the other rules satisfy Independence of Losing Alternatives.

- *First Majority*: Assume *E* is an election such that $$ sc (E) = (3,2,1,0)$$. Then the winner set under *First Majority* is $$\{c_1,c_2\}$$ but removing $$c_3$$ changes the winner set to $$\{c_1\}$$.
- *Largest Gap*: Consider the same election as for *First Majority*. Then, the winner set under *Largest Gap* is $\{c_1\}$ but removing $c_3$ changes this to $\{c_1,c_2\}$.
- *Next*-$$k$$: Consider an election *E* with $$ sc (E) = (4,3,2,0)$$. Then, for every $$k > 1$$, we have $$\mathcal {R}(E) = \{c_1,c_2\}$$ under *Next*-$$k$$, but after deleting $$c_3$$ we have $$\mathcal {R}(E) = \{c_1\}$$.

Consider, e.g., the class of *Size Priority* instances defined by any order of the form $$2 \vartriangleright 1 \vartriangleright \dots $$ and an election *E* with $$ sc (E) = (2,1,1)$$. Then $$\mathcal {R}(E) = \{c_1\}$$ but the removal of $$c_3$$ leads to $$\mathcal {R}(E) = \{c_1,c_2\}$$. Thus, *Size Priority* fails Independence of Losing Alternatives in general. However, we claim that every class of *Increasing Size Priority* instances satisfies Independence of Losing Alternatives. We distinguish two cases: First assume all *m*-alternatives are selected. Then Independence of Losing Alternatives is vacuously satisfied as there are no losing alternatives. On the other hand, assume that there is a $$k < m$$ such that $$\{c_1, \dots ,c_k\}$$ is winning. As $$\mathcal {R}$$ is an *Increasing Size Priority* instance there is a $$k' \le k$$ such that $$\vartriangleright $$ restricted to $$\{1, \dots , m\}$$ starts with $$k' \vartriangleright k'+1 \vartriangleright \dots \vartriangleright k$$. As $$k < m$$ the same holds for $$\vartriangleright $$ restricted to $$\{1, \dots , m-1\}$$. Hence if we remove an alternative $$c_{k^*}$$ with $$k^* > k$$ the winner set does not change.

Finally, we claim that *Top*-$s$-*First*-$k$-*Gap* also satisfies Independence of Losing Alternatives. Assume first that the set of winners $W'$ under *First* $k$-*Gap* is smaller than *s*. After removing a losing alternative, the set of winners under *First* $k$-*Gap* remains the same and is hence still smaller then *s*. It follows that the winner set under *Top*-$s$-*First*-$k$-*Gap* does not change. Now assume that $W'$ is larger than *s*. Then, the winner set of *Top*-$s$-*First*-$k$-*Gap* has size at least *s*. Now, removing an alternative $c_j$ for $j > s$ cannot create a larger gap between the first *s* alternatives. It follows that the winner set under *First* $k$-*Gap* after removing $c_j$ is still larger then *s*. This means by definition that the winner set of *Top*-$s$-*First*-$k$-*Gap* before and after removing $c_j$ was the winner set of *Increasing Size Priority*. As *Increasing Size Priority* satisfies Independence of Losing Alternatives, we can conclude that *Top*-$s$-*First*-$k$-*Gap* does so as well.

#### Resistance to clones

Clearly, Independence implies Resistance to Clones. Hence, $f$-*Threshold* satisfies Resistance to Clones. As cloning does not change the maximal score, the same holds for *Max-Score*-$f$-*Threshold*. Furthermore, cloning has no effect on gaps, hence *Largest Gap* and *First* $k$-*Gap* satisfy Resistance to Clones. If follows that *Approval Voting* also satisfies Resistance to Clones as it is a special case of *First* $k$-*Gap*.

For *First Majority* and *Next*-$$k$$ it can be helpful for an alternative to be cloned. For example, consider an election with $$ sc _E = (3,2,0)$$. Then the set of *First Majority* winners would be $$\{c_1\}$$ but after cloning $$c_2$$, the set of *First Majority* winners is $$\{c_1,c_2,c_2'\}$$ where $$c_2'$$ is the clone of $$c_2$$. Similarly let $$ sc _E = (2,1,0)$$. Then the set of *Next*-$$k$$ winners for every *k* would be $$\{c_1\}$$. If we clone $$c_2$$, then, for all $$k \ge 2$$, *Next*-$$k$$ selects $$\{c_1,c_2,c_2'\}$$ where $$c_2'$$ is the clone of $$c_2$$.

Moreover, *q-NCSA* also does not satisfy Resistance to Clones. Consider for example 0.5-*NCSA*, assume we have 10 voters and let $$ sc _E = (10,7,7)$$. Then the 0.5-*NCSA*-scores of $$\{c_1\}$$, $$\{c_1,c_2\}$$ and $$\{c_1,c_2,c_3\}$$ are 10/1, $${14}/{\sqrt{2}} \approx 9.899$$ and $${18}/{\sqrt{3}} \approx 10.392$$ respectively. Therefore $$\{c_1,c_2,c_3\}$$ is the winner set. Now, if we add a clone $$c_1^*$$ of $$c_1$$ we get the 0.5-*NCSA*-scores 10/1, $${20}/{\sqrt{2}} \approx 14.142$$, $${24}/{\sqrt{3}} \approx 13.856$$ and $${28}/{\sqrt{4}} = 14$$ for $$\{c_1\}$$, $$\{c_1, c_1^*\}$$, $$\{c_1, c_1^*,c_2\}$$ and $$\{c_1, c_1^*,c_2,c_3\}$$ respectively. Therefore, $$\{c_1, c_1^*\}$$ is winning.

Finally, *Size Priority* generally does not satisfy Resistance to Clones as cloning may harm an alternative. For example, consider $$2 \vartriangleright 3 \vartriangleright \dots $$ and $$ sc _E = (2,1,0)$$. Then the set of *Size Priority* winners is $$\{c_1, c_2\}$$, but if we clone $$c_1$$, then $$c_2$$ is not a winner any more. This also shows that neither *Increasing Size Priority* nor *Top*-$$s$$-*First*-$$k$$-*Gap* are resistant to clones (set $$s= 2$$ and $$k=2$$ for the latter).

#### Set monotonicity

- Let *E* be an election with $$ sc (E) = (2,2,1,1,1,1)$$. Then under *First Majority* we have $$\mathcal {R}(E) = \{c_1, c_2,c_3\}$$. Now if a voter who did not approve $$\{c_1, c_2,c_3\}$$ before approves it, then we get $$ sc (E) = (3,3,2,1,1,1)$$ and hence $$\mathcal {R}(E) = \{c_1, c_2\}$$.
- For *Max-Score*-$f$-*Threshold* first assume
  $$\begin{aligned}f(n):= {\left\{ \begin{array}{ll} n-1 &{}\text { if } n \text { is odd},\\ 1 &{}\text { otherwise}. \end{array}\right. } \end{aligned}$$
  Let $$ sc (E) = (5, 2)$$. Then $$c_1$$ is the only winner, but after adding one approval to $$c_1$$ the winner set becomes $$\{c_1,c_2\}$$. Now, assume $$f(n) = \alpha \cdot n$$ for some $$0 \le \alpha < 1$$. First assume $$ sc _E(c_i) > \alpha \max ( sc (E))$$ and hence $$c_i \in \mathcal {R}(E)$$. Then after adding one approval to all winning candidates, we have
  $$\begin{aligned} sc _{E^*}(c_i)= sc _E(c_i) + 1> \alpha \max ( sc (E)) +1 \ge \\ \alpha (\max ( sc (E)) +1) = \alpha \max ( sc (E^*)). \end{aligned}$$
  This implies $c_i \in \mathcal {R}(E^*)$. On the other hand, assume $sc _E(c_i) \le \alpha \max ( sc (E))$ and hence $c_i \not \in \mathcal {R}(E)$. Then $sc _{E^*}(c_i)= sc _E(c_i) < \alpha (\max ( sc (E))) \le \alpha \max ( sc (E^*))$. It follows that $c_i \not \in \mathcal {R}(E^*)$. Therefore, *Max-Score*-$f$-*Threshold* satisfies Set Monotonicity for constant *f*.
- Clearly, adding approvals for all winners can only increase the gap between winners and non-winners. Hence *First* $k$-*Gap* and *Largest Gap* satisfy Set Monotonicity. *Approval Voting* is a special case of *First* $k$-*Gap* and hence also satisfies Set Monotonicity.
- For $f$-*Threshold* clearly all winning candidates are still above the threshold in $E^*$ and all non-winning candidates remain below the threshold. Hence Set Monotonicity is satisfied.
- *Size Priority*: It is easy to see that a set $\{c_1, \dots , c_i\}$ is non-tiebreaking in *E* if and only if it is non-tiebreaking in $E^*$. Hence, *Size Priority* satisfies Set Monotonicity.
- *Next*-$k$: Let $\{c_1, \dots , c_{\ell }\}$ be the winner set. First, let $i < \ell$. By choice of $\ell$ we have $sc _E(c_i) \le \sum _{j = 1}^k sc _E(c_{i+j})$. As $i +1 \le \ell$ we have $sc _{E^*}(c_i) = sc _E(c_i) +1 \le \sum _{j = 1}^k sc _E(c_{i+j}) + 1 \le \sum _{j = 1}^k sc _{E^*}(c_{i+j})$. On the other hand $sc _{E^*}(c_{\ell })> sc _E(c_{\ell }) > \sum _{j = 1}^k sc _E(c_{{\ell }+j}) = \sum _{j = 1}^k sc _{E^*}(c_{{\ell }+j})$. It follows that $\{c_1, \dots , c_{\ell }\}$ is also the winner set under $E^*$.
- *q-NCSA*: Let $W=\{c_1, \dots , c_k\}$ be a the largest set with maximum *q-NCSA*-score. It holds that
  $$\begin{aligned} sc _{E^*}^{\textit{q-NCSA}}(W)&=\frac{1}{|W|^q}\sum _{c \in W}(2 sc _{E^*}(c) - n)=\\&=\frac{1}{|W|^q}\sum _{c \in W}(2( sc _E(c) + 1) - n)=\\&= sc _{E}^{\textit{q-NCSA}}(W) + \frac{2|W|}{|W|^q}\\&= sc _{E}^{\textit{q-NCSA}}(W) + 2|W|^{1-q} \end{aligned}$$
  Now consider a set $W'=\{c_1, \dots , c_i\}$. Consider first $i < k$. Then we have by the same argument as above
  $$\begin{aligned} sc _{E^*}^{\textit{q-NCSA}}(W')= sc _{E}^{\textit{q-NCSA}}(W') + 2|W'|^{1-q} \end{aligned}$$
  Now, by the choice of *k* we have $sc _E^{\textit{q-NCSA}}(W') \le sc _E^{\textit{q-NCSA}}(W')$ and because $i < k$ we have $|W' |< |W |$. It follows that $sc _{E^*}^{\textit{q-NCSA}}(W') \le sc _{E^*}^{\textit{q-NCSA}}(W')$. Now assume $k < i$. Then we have
  $$\begin{aligned} sc _{E^*}^{\textit{q-NCSA}}(W')&=\frac{1}{|W'|^q}\left( \sum _{j \le k}(2( sc _E(c_j) + 1) - n\right) + \sum _{j > k}(2 sc _E(c_j) - n)\\&= sc _{E}^{\textit{q-NCSA}}(W') + \frac{2|W|}{|W'|^q} \end{aligned}$$
  Again, by the choice of *k* we have $sc _E^{\textit{q-NCSA}}(W') \le sc _E^{\textit{q-NCSA}}(W')$. Moreover, because $i > k$ we have $|W' |> |W |$ and hence
  $$\begin{aligned}\frac{2|W|}{|W' |^q} < \frac{2|W|}{|W|^q}\end{aligned}$$
  It follows that $sc _{E^*}^{\textit{q-NCSA}}(W') \le sc _{E^*}^{\textit{q-NCSA}}(W')$ and hence *W* is still the largest set with maximal *q-NCSA*-score.
- Finally, consider *Top*-$s$-*First*-$k$-*Gap*. Assume first that the set of winners $W'$ under *First* $k$-*Gap* is smaller than *s*. As *First* $k$-*Gap* satisfies Set Monotonicity, $W'$ remains the winner set in $E^*$. It is still smaller than *s* and therefore still the winner under *Top*-$s$-*First*-$k$-*Gap*. Now assume that $W'$ is larger than *s*. Then, the winner set of *Top*-$s$-*First*-$k$-*Gap* has size at least *s*. Adding one approval to the first *s* alternatives does not create a new *k* gap between them. It follows that the winner set under *First* $k$-*Gap* is still larger then *s*. This means by definition that the winner set of *Top*-$s$-*First*-$k$-*Gap* in *E* and $E^*$ is the winner set of *Increasing Size Priority*. As *Increasing Size Priority* satisfies Set Monotonicity, we can conclude that *Top*-$s$-*First*-$k$-*Gap* does so as well.

#### Superset monotonicity

It was shown in the main text that *Increasing Size Priority*, *First* $k$-*Gap* and *Top*-$s$-*First*-$k$-*Gap* satisfy Superset Monotonicity. Let us now show that *First Majority*, $f$-*Threshold*, *Max-Score*-$f$-*Threshold*, *Next*-$k$, *Largest Gap* and *Size Priority* do not satisfy Superset Monotonicity.

- Clearly, Superset Monotonicity implies Set Monotonicity, hence *First Majority* cannot satisfy Superset Monotonicity.
- First, consider an election *E* with $$n= 3$$ such that $$ sc (E) = (2,1)$$. Then $$\mathcal {R}(E) = \{c_1\}$$ under $$f$$-*Threshold* with $$f ={n}/{2}$$. Now, if one voter additionally approves $$\{c_1,c_2\}$$, then $$\mathcal {R}(E) = \{c_1,c_2\}$$.
- Next, consider an election *E* such that $$ sc (E) = (4,2)$$. Consider *Max-Score*-$$f$$-*Threshold* with $$f(n) = {n}/{2}$$. Then $$\mathcal {R}(E) = \{c_1\}$$. Now, if one voter additionally approves $$\{c_1,c_2\}$$, then $$\mathcal {R}(E) = \{c_1,c_2\}$$.
- For *Next*-$$k$$, consider an election *E* such that $$ sc (E) = (3,1,1)$$. Then the winner set under *Next*-$$k$$ is $$\{c_1\}$$. Now, if a voter changes her mind and additionally approves all three alternatives, then all three alternatives become winners under *Next*-$$k$$ (for every $$k>1$$).
- Next, consider an election *E* such that $$ sc (E) = (2,1,0)$$. For *Largest Gap*, $$\mathcal {R}(E) = \{c_1\}$$. If one voter additionally approves $$\{c_1,c_2\}$$, then $$ sc (E) = (3,2,0)$$ and $$\mathcal {R}(E) = \{c_1,c_2\}$$.
- For *Size Priority*, consider an election *E* with $$ sc (E) = (2,1,1)$$ and $$2 \vartriangleright 1 \vartriangleright 3 \vartriangleright 0$$. Then $$\mathcal {R}(E) = \{c_1\}$$. Now, if one voter additionally approves $$\{c_1,c_2\}$$, then $$\mathcal {R}(E) = \{c_1,c_2\}$$.
- For 0.5-*NCSA*, consider an election *E* with $$ sc (E) = (90,90,67)$$ and $$n=98$$. Here, the winner set is $$\{c_1, c_2\}$$ with $$ sc _{E}^{\textit{q-NCSA}}(\{c_1,c_2\})\approx 115.97$$ and $$ sc _{E}^{\textit{q-NCSA}}(\{c_1,c_2, c_3\})\approx 115.47$$. However, for $$ sc (E^*) = (91,91,68)$$ (one voter who previously approved no one, now approves every candidate), we obtain $$ sc _{E^*}^{\textit{q-NCSA}}(\{c_1,c_2\})\approx 118.79$$ and $$ sc _{E^*}^{\textit{q-NCSA}}(\{c_1,c_2, c_3\})\approx 118.93$$.

### C. Proof of Claim 1 in Proposition 1

**Claim** 1 Let *E* be an election. Then if $$ sc _E(c_i) = sc _E(c_{i+1})$$ and

$$\begin{aligned} sc _E^{\textit{q-NCSA}}(\{c_1, \dots , c_{i-1}\}) \le sc _E^{\textit{q-NCSA}}(\{c_1, \dots , c_{i}\})\end{aligned}$$

then also

$$\begin{aligned} sc _E^{\textit{q-NCSA}}(\{c_1, \dots , c_{i}\}) \le sc _E^{\textit{q-NCSA}}(\{c_1, \dots , c_{i+1}\}).\end{aligned}$$

#### Proof

Assume $$ sc _E(c_i) = sc _E(c_{i+1})$$ and $$ sc _E^{\textit{q-NCSA}}(\{c_1, \dots , c_{i-1}\}) \le sc _E^{\textit{q-NCSA}}(\{c_1, \dots , c_{i}\})$$. To enhance readability, we write *x* for $$\sum _{k=1}^{i-1}(2 sc _E(c_k) - n)$$ and *y* for $$2 sc _E(c_i) - n$$. Then, we can write

$$\begin{aligned} sc _E^{\textit{q-NCSA}}(\{c_1, \dots , c_{i-1}\}) \le sc _E^{\textit{q-NCSA}}(\{c_1, \dots , c_{i}\})\end{aligned}$$

as

$$\begin{aligned}\frac{x}{|\{c_1, \dots ,c_{i-1}\}|^q} =\frac{x}{(i-1)^q} \le \frac{x+y}{i^q} = \frac{x+y}{|\{c_1, \dots ,c_{i}\}|^q}\end{aligned}$$

Now, defining *z* as $i^q - (i-1)^q$, we can rewrite this as

$$\begin{aligned}\frac{x}{(i-1)^q} \le \frac{x+y}{(i-1)^q + (i^q - (i-1)^q)} = \frac{x+y}{(i-1)^q + z}\end{aligned}$$

Then we can do the following computation:

$$\begin{aligned} \frac{x}{(i-1)^q}&\le \frac{x+y}{(i-1)^q + z}\\ x((i-1)^q+z)&\le (x+y)(i-1)^q\\ x(i-1)^q+xz&\le x(i-1)^q + y(i-1)^q\\ xz&\le y(i-1)^q\\ xz +yz&\le y(i-1)^q + yz\\ z(x +y)&\le y((i-1)^q + z)\\ z\frac{x+y}{(i-1)^q +z}&\le y\\ x+y +z\frac{x+y}{(i-1)^q +z}&\le x+y+y\\ \frac{(x+y)((i-1)^q+z)}{(i-1)^q+z} +z\frac{x+y}{(i-1)^q +z}&\le x+2y\\ \frac{x+y}{(i-1)^q+z}((i-1)^q+2z)&\le x+2y\\ \frac{x+y}{(i-1)^q+z}&\le \frac{x+2y}{((i-1)^q+2z)}\\ \end{aligned}$$

Now replacing *x*, *y*, *z* again by their respective definition we get for the left-hand side:

$$\begin{aligned} \frac{\sum _{k=1}^{i-1}(2 sc _E(c_k) - n) + (2 sc _E(c_i) - n)}{(i-1)^q +(i^q - (i-1)^q)}&= \frac{\sum _{k=1}^{i}(2 sc _E(c_k) - n)}{i^q} \\ {}&= sc _E^{\textit{q-NCSA}}(\{c_1, \dots , c_{i}\}) \end{aligned}$$

Observe that, by definition, we have $y =2 sc _E(c_{i}) - n = 2 sc _E(c_{i+1}) - n$. Therefore, we can write the right-hand side as

$$\begin{aligned}\frac{\sum _{k=1}^{i-1}(2 sc _E(c_k) - n) + (2 sc _E(c_i) - n) + (2 sc _E(c_{i+1}) - n)}{(i-1)^q +(i^q - (i-1)^q) + z} = \frac{\sum _{k=1}^{i+1}(2 sc _E(c_k) - n)}{i^q + z} \end{aligned}$$

Now, we claim that because $0 \le q \le 1$ we have

$$\begin{aligned}z = i^q - (i-1)^q \ge (i+1)^q - i^q.\end{aligned}$$

We observe that the both sides of the equation equal the change of the function $x^q$ in an interval of one. Because the derivative of $f(x)=x^q$ for $0 \le q \le 1$ is monotone declining, we can bound this change using the slope of *f*(*x*) in either the starting or end point of the interval as follows

$$\begin{aligned}1 \cdot f'(x+1) \le f(x+1) - f(x) \le 1 \cdot f'(x).\end{aligned}$$

Therefore, we have

$$\begin{aligned}i^q - (i-1)^q \ge f'(i) \ge (i+1)^q - i^q\end{aligned}$$

It follows that

$$\begin{aligned} \frac{\sum _{k=1}^{i+1}(2 sc _E(c_k) - n)}{i^q + z} \le \frac{\sum _{k=1}^{i+1}(2 sc _E(c_k) - n)}{i^q + (i+1)^q - i^q} = sc _E^{\textit{q-NCSA}}(\{c_1, \dots , c_{i+1}\}).\end{aligned}$$

All together we have shown

$$\begin{aligned} sc _E^{\textit{q-NCSA}}(\{c_1, \dots , c_{i}\}) \le sc _E^{\textit{q-NCSA}}(\{c_1, \dots , c_{i+1}\}).\end{aligned}$$

This concludes the proof. $\square$

## Footnotes

1 For the same reason, approval ballots are also used for crowd-sourced labeling of training data for machine-learning purposes (Procaccia and Shah 2015; Shah and Zhou 2020; Allouche et al. 2022a).

2 Faliszewski et al. (2020) consider $f$-*Threshold* (under the name *NAV* and with a different definition that is equivalent up to tiebreaking), *Approval Voting* (under the name *NCSA* and with a different definition that is equivalent up to tiebreaking), *First Majority* and *q-NCSA*.

3 In the following, we use the words candidate and alternative interchangeably.

4 The given definition is different than the original definition of the *q-NCSA*-score by Faliszewski et al. (2020). However, it can be shown that both definitions are equivalent. See Appendix A for more details.

5 In the original definition of Faliszewski et al. (2020), all sets with maximum *q-NCSA*-score are co-winners. We specifically select the largest set with maximum *q-NCSA*-score as this choice guarantees that the winner set is non-tiebreaking (see Proposition 1).

6 This is not equivalent to $f$-*Threshold* for $f(n) = \frac{1}{2}n$, as candidates with exactly $\frac{1}{2}n$ approval score are not included in the winner set of $f$-*Threshold*.

7 The relation between approval voting and maximum likelihood estimation is analyzed in detail by Procaccia and Shah (2015), in particular, under which conditions approval voting selects the most likely “best” alternatives.

8 We make this assumption because a bias *only* against low-quality alternatives is actually helpful for the shortlisting task—this effect would distort the negative consequences of bias.

9 We briefly describe “E Pluribus Hugo”. This is an approval-based variable multi-winner rule based on an elimination process with two scores: approval scores and fractional approval scores. Let $N_E(c)=\{i\in N: c\in v_i\}$. Fractional approval scores are defined as $fsc _E(c_j)=\sum _{i\in N_E(c_j)} \frac{1}{|v_i|}$, i.e., voters can contribute at most 1 to the total score of all candidates. Each round the two candidates with the lowest fractional approval scores are selected. Out of these two, the one with the lower approval score is eliminated. This step is repeated with a reduced set of candidates (and updated fractional approval scores) until only six candidates remain. We omit details how ties are handled in this process and refer to Quinn and Schneier (2016), who introduced “E Pluribus Hugo” under the name SDV-LPE. This paper also contains a discussion of why this rule was chosen (in reaction to strategic voting in previous years) and its merits for this specific application.

10 One could even use these two metrics to define shortlisting rules as a bicriteria optimization problem, i.e., to find a shortlist that has a large precision and small size. Note, however, that this approach requires two non-trivial choices: (i) a function estimating precision based on $$ sc _{E}$$, and (ii) a method for balancing these two objectives (e.g., by weighting precision and size). While this approach is interesting, we view it as impractical due to these rather technical choices.

11 The approach of Esmaeili et al. (2021), i.e., to impose bounds on the “price of fairness”, could be an interesting strategy to find suitable tradeoffs between efficiency and fairness.

## References

[CR1] Allouche T, Lang J, Yger F (2022a) Multi-winner approval voting goes epistemic. In: The 38th conference on uncertainty in artificial intelligence (UAI 2022)

[CR2] Allouche T, Lang J, Yger F (2022b) Truth-tracking via approval voting: size matters. In: Proceedings of the 36th conference on artificial intelligence (AAAI-2022), pp 4768–4775

[CR3] Amegashie JA (1999) The design of rent-seeking competitions: committees, preliminary and final contests. Public Choice 99(1–2):63–76

[CR4] Aziz H, Brill M, Conitzer V et al (2017a) Justified representation in approval-based committee voting. Soc Choice Welfare 48(2):461–485

[CR5] Aziz H, Elkind E, Faliszewski P et al (2017b) The Condorcet principle for multiwinner elections: from shortlisting to proportionality. In: Proceedings of the 26th international joint conference on artificial intelligence (IJCAI-2017), pp 84–90

[CR6] Barberà S, Coelho D (2017) Balancing the power to appoint officers. Games Econom Behav 101:189–203

[CR7] Barberà S, Coelho D (2022) Compromising on compromise rules. Rand J Econ 53(1):95–112

[CR8] Bovens L (2016) Selection under uncertainty: affirmative action at shortlisting stage. Mind 125(498):421–437

[CR9] Brams SJ, Fishburn PC (1978) Approval voting. Am Polit Sci Rev 72(3):831–847

[CR10] Brams SJ, Kilgour M (2012) Narrowing the field in elections: the next-two rule. J Theor Polit 24(4):507–525

[CR11] Brams SJ, Kilgour M (2015) Satisfaction Approval Voting. In: Melnik R (ed) Mathematical and Computational Modeling, pp 273–298

[CR12] Brandl F, Peters D (2019) An axiomatic characterization of the Borda mean rule. Soc Choice Welfare 52(4):685–707 31057193 10.1007/s00355-018-1167-8 PMC6472553

[CR13] Bredereck R, Kaczmarczyk A, Niedermeier R (2017) On coalitional manipulation for multiwinner elections: shortlisting. In: Proceedings of the 26th international joint conference on artificial intelligence (IJCAI-2017), pp 887–893

[CR14] Bredereck R, Faliszewski P, Kaczmarczyk A et al (2019) An experimental view on committees providing justified representation. In: Proceedings of the 28th international joint conference on artificial intelligence (IJCAI-2019), pp 109–115

[CR15] BWAA B (2019) BBWAA election rules. https://baseballhall.org/hall-of-famers/rules/bbwaa-rules-for-election. Accessed 12 November 2019

[CR16] Chhabra A, Masalkovaite K, Mohapatra P (2021) An overview of fairness in clustering. IEEE Access 9:130698–130720

[CR17] de Clippel G, Eliaz K, Knight B (2014) On the selection of arbitrators. Am Econ Rev 104(11):3434–3458

[CR18] Duddy C, Houy N, Lang J et al (2014) Social dichotomy functions, extended abstract for presentation at the 2014 meeting of the Society for Social Choice and Welfare

[CR19] Duddy C, Piggins A, Zwicker WS (2016) Aggregation of binary evaluations: a Borda-like approach. Soc Choice Welfare 46(2):301–333

[CR20] Dutta R, Horan S (2015) Inferring rationales from choice: identification for rational shortlist methods. Am Econ J Microecon 7(4):179–201

[CR21] Elkind E, Slinko A (2016) Rationalizations of voting rules. In: Brandt F, Conitzer V, Endriss U et al (eds) Handbook of computational social choice, 1st edn. Cambridge University Press, Cambridge, pp 169–196

[CR22] Elkind E, Faliszewski P, Laslier JF, et al (2017a) What do multiwinner voting rules do? An experiment over the two-dimensional Euclidean domain. In: Proceedings of the 31st conference on artificial intelligence (AAAI-2017). AAAI Press, pp 494–501

[CR23] Elkind E, Faliszewski P, Skowron P et al (2017b) Properties of multiwinner voting rules. Soc Choice Welfare 48(3):599–632 32226187 10.1007/s00355-017-1026-z PMC7089675

[CR24] Endriss U (2016) Judgment aggregation. In: Brandt F, Conitzer V, Endriss U et al (eds) Handbook of computational social choice, 1st edn. Cambridge University Press, Cambridge, pp 399–426

[CR25] Esmaeili S, Brubach B, Srinivasan A et al (2021) Fair clustering under a bounded cost. Adv Neural Inf Process Syst 34:14345–14357

[CR26] Faliszewski P, Skowron P, Slinko A et al (2017) Multiwinner voting: a new challenge for social choice theory. In: Endriss U (ed) Trends in computational social choice, chap 2. AI Access, pp 27–47

[CR27] Faliszewski P, Slinko A, Talmon N (2020) The complexity of multiwinner voting rules with variable number of winners. In: Proceedings of the 24th European conference on artificial intelligence (ECAI-2020)

[CR28] Fernández LS, Elkind E, Lackner M et al (2017) Proportional justified representation. In: Proceedings of the 31st conference on artificial intelligence (AAAI-2017). AAAI Press, pp 670–676

[CR29] Freeman R, Kahng A, Pennock DM (2020) Proportionality in approval-based elections with a variable number of winners. In: Proceedings of the 29th international joint conference on artificial intelligence (IJCAI-2020), pp 132–138

[CR30] Gangl C, Lackner M, Maly J et al (2019) Aggregating expert opinions in support of medical diagnostic decision-making. In: Knowledge representation for health care/prohealth (KR4HC), pp 56–62

[CR31] Horan S (2016) A simple model of two-stage choice. J Econ Theory 162:372–406

[CR32] Kilgour M (2010) Approval balloting for multi-winner elections. In: Laslier JF, Sanver R (eds) Handbook on approval voting. Springer, Berlin, pp 105–124

[CR33] Kilgour M (2016) Approval elections with a variable number of winners. Theory Decision 81:199–211

[CR34] Kilgour M, Marshall E (2012) Approval balloting for fixed-size committees. Electoral systems. Springer, Berlin, pp 305–326

[CR35] Klemperer P (1999) Auction theory: a guide to the literature. J Econ Surv 13(3):227–286

[CR36] Kops C (2018) (F)lexicographic shortlist method. Econ Theor 65(1):79–97

[CR37] Lackner M, Maly J (2021) Approval-based shortlisting. In: Proceedings of the 20th international conference on autonomous agents and multiagent systems (AAMAS-2021). IFAAMAS, pp 1566–1568

[CR38] Lackner M, Maly J (2022) Python code for ”approval-based shortlisting”. 10.5281/zenodo.3821983 PMC11772499 39882568

[CR39] Lackner M, Skowron P (2021) Consistent approval-based multi-winner rules. J Econ Theory 192(105):173

[CR40] Lackner M, Skowron P (2023) Multi-winner voting with approval preferences. Springer, Berlin

[CR41] List C (2012) The theory of judgment aggregation: an introductory review. Synthese 187(1):179–207

[CR42] Manzini P, Mariotti M (2007) Sequentially rationalizable choice. Am Econ Rev 97(5):1824–1839

[CR43] Núñez M, Laslier JF (2015) Bargaining through approval. J Math Econ 60:63–73

[CR44] Procaccia AD, Shah N (2015) Is approval voting optimal given approval votes? In: Advances in neural information processing systems, pp 1801–1809

[CR45] Quinn J, Schneier B (2016) A proportional voting system for awards nominations resistant to voting blocs. Preprint per https://www.schneier.com/academic/archives/2016/05/a_proportional_votin.html. Accessed 14 November 2019

[CR46] Rey S, Endriss U, de Haan R (2021) Shortlisting rules and incentives in an end-to-end model for participatory budgeting. In: Proceedings of the 30th international joint conference on artificial intelligence (IJCAI-2021), pp 370–376

[CR47] Sánchez-Fernández L, Fisteus JA (2019) Monotonicity axioms in approval-based multi-winner voting rules. In: Proceedings of the 18th international conference on autonomous agents and multiagent systems (AAMAS-2019). International Foundation for Autonomous Agents and Multiagent Systems, pp 485–493

[CR48] Shah NB, Zhou D (2020) Approval voting and incentives in crowdsourcing. ACM Trans Econ Comput (TEAC) 8(3):1–40

[CR49] Shalev-Shwartz S, Ben-David S (2014) Understanding machine learning: from theory to algorithms. Cambridge University Press, Cambridge

[CR50] Shoham Y, Leyton-Brown K (2009) Multiagent systems—algorithmic, game-theoretic, and logical foundations. Cambridge University Press, Cambridge

[CR51] Singh A, Rose C, Visweswariah K et al (2010) Prospect: a system for screening candidates for recruitment. In: Proceedings of the 19th ACM international conference on information and knowledge management. ACM, pp 659–668

[CR52] The Hugo Awards (2019) The voting system. http://www.thehugoawards.org/the-voting-system. Accessed 12 November 2019

[CR53] The Man Booker Prize (2018) Rules & entry form. https://thebookerprizes.com/sites/manbosamjo/files/uploadedfiles/files/ManBookerPrize2018RulesAndEntryForm.pdf. Accessed 13 November 2019

[CR54] Tideman TN (1987) Independence of clones as a criterion for voting rules. Soc Choice Welfare 4(3):185–206

[CR55] Tweeddale HM, Cameron RF, Sylvester SS (1992) Some experiences in hazard identification and risk shortlisting. J Loss Prev Process Ind 5(5):279–288

[CR56] Tyson CJ (2013) Behavioral implications of shortlisting procedures. Soc Choice Welfare 41(4):941–963

[CR57] Zwicker WS, Moulin H (2016) Introduction to the theory of voting. In: Brandt F, Conitzer V, Endriss U et al (eds) Handbook of computational social choice. Cambridge University Press, Cambridge, pp 23–56

