# Supplementary figures and images for: Approval-based shortlisting
Source: Soc Choice Welfare. 2023 Aug 11;64(1-2):97–142. doi: 10.1007/s00355-023-01482-2 (PMC11772499; doi:10.1007/s00355-023-01482-2)

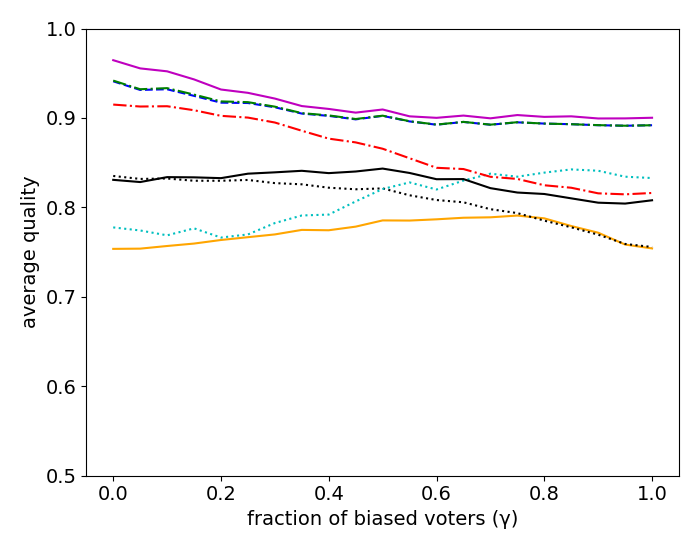

Supplement: Supplementary file 1 — (png 51 KB) [file 355_2023_1482_MOESM1_ESM.png]

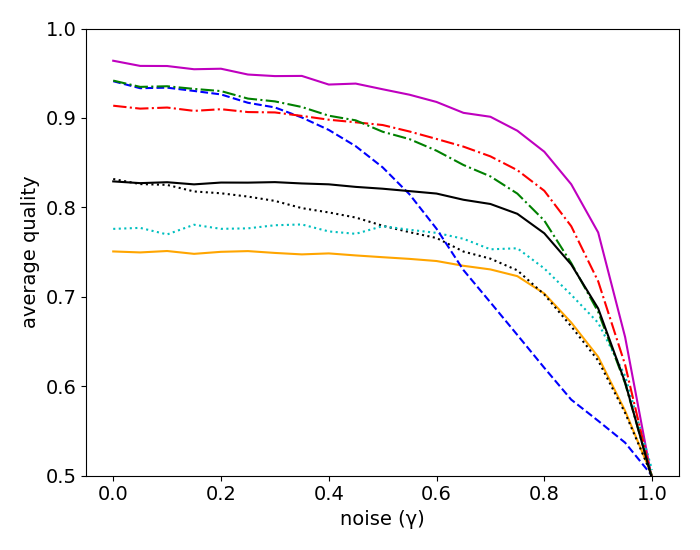

Supplement: Supplementary file 2 — (png 46 KB) [file 355_2023_1482_MOESM2_ESM.png]

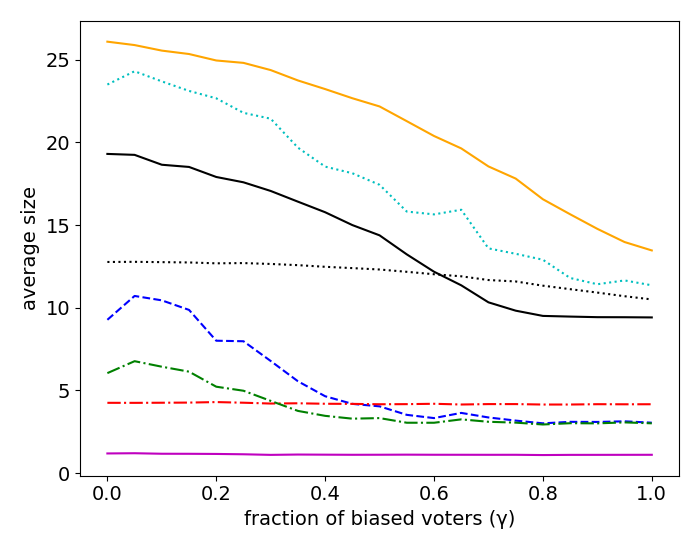

Supplement: Supplementary file 3 — (png 60 KB) [file 355_2023_1482_MOESM3_ESM.png]

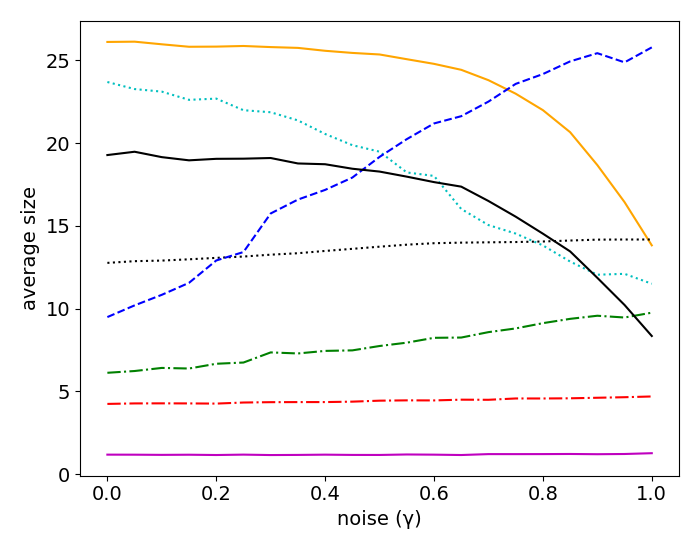

Supplement: Supplementary file 4 — (png 51 KB) [file 355_2023_1482_MOESM4_ESM.png]

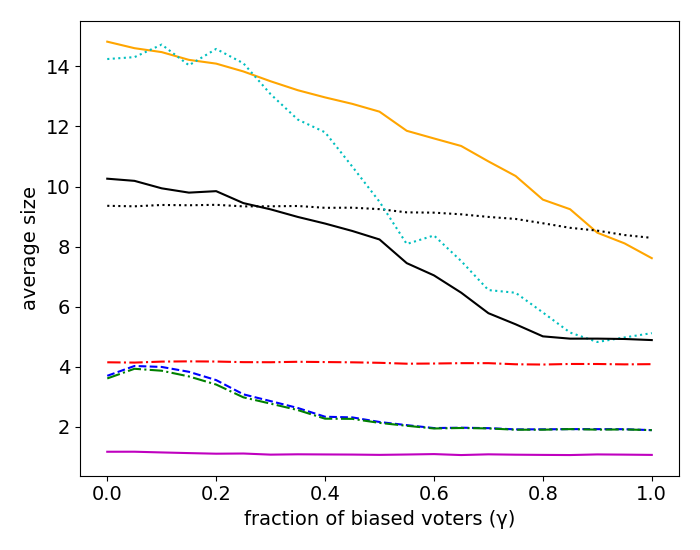

Supplement: Supplementary file 5 — (png 47 KB) [file 355_2023_1482_MOESM5_ESM.png]

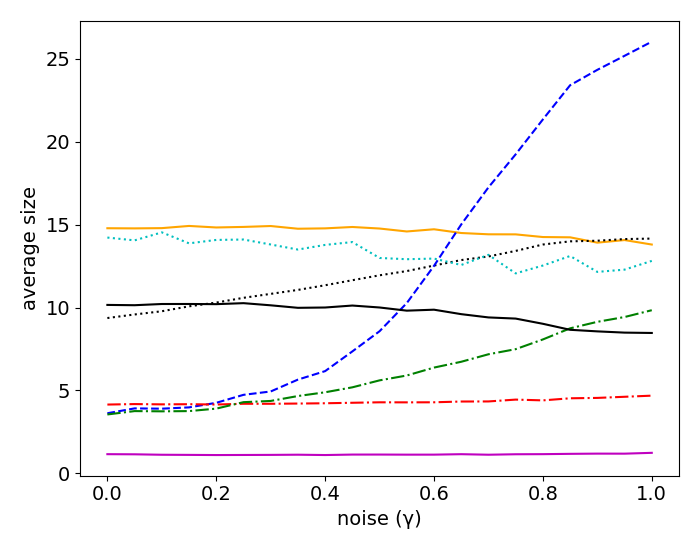

Supplement: Supplementary file 6 — (png 50 KB) [file 355_2023_1482_MOESM6_ESM.png]

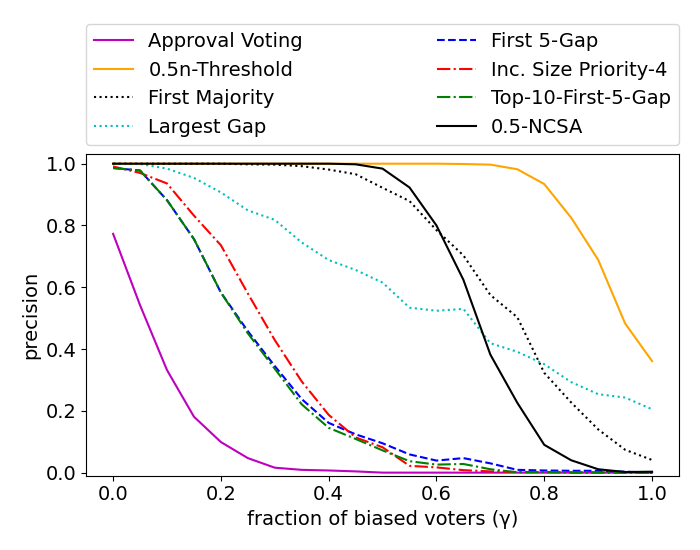

Supplement: Supplementary file 7 — (png 43 KB) [file 355_2023_1482_MOESM7_ESM.png]

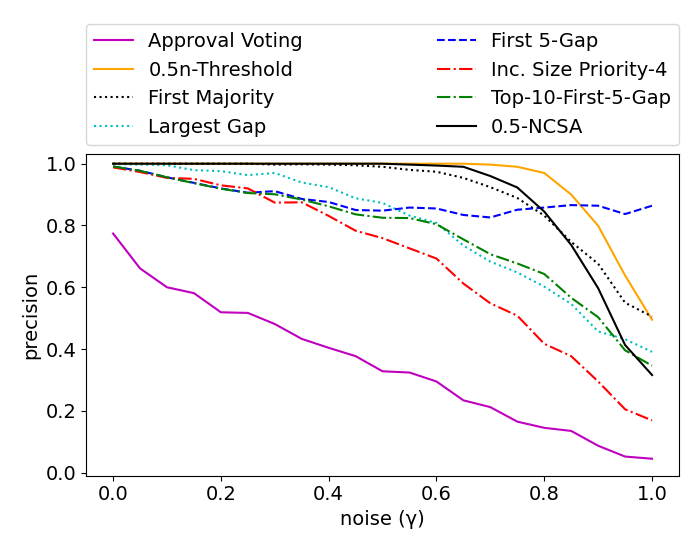

Supplement: Supplementary file 8 — (png 76 KB) [file 355_2023_1482_MOESM8_ESM.png]

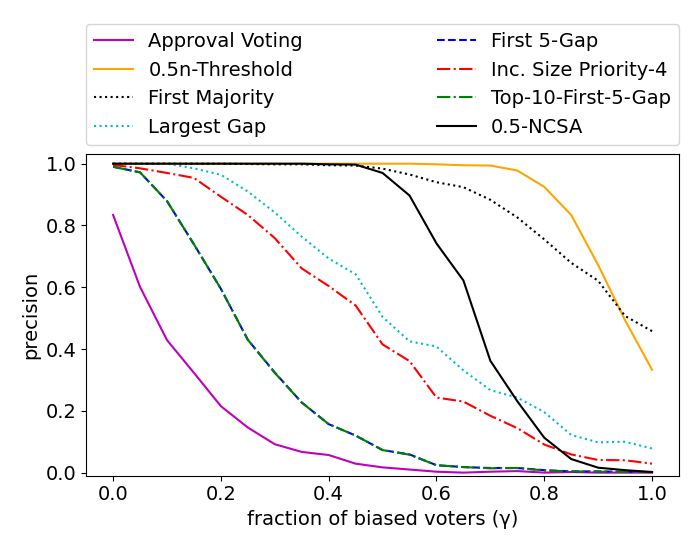

Supplement: Supplementary file 9 — (png 71 KB) [file 355_2023_1482_MOESM9_ESM.png]

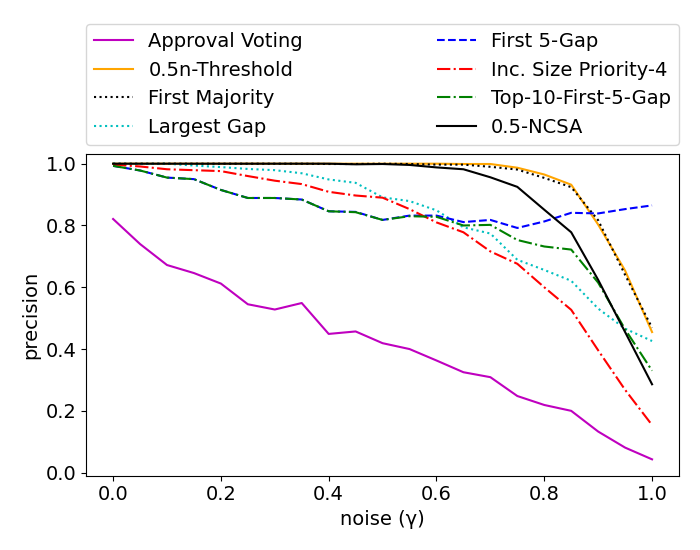

Supplement: Supplementary file 10 — (png 76 KB) [file 355_2023_1482_MOESM10_ESM.png]

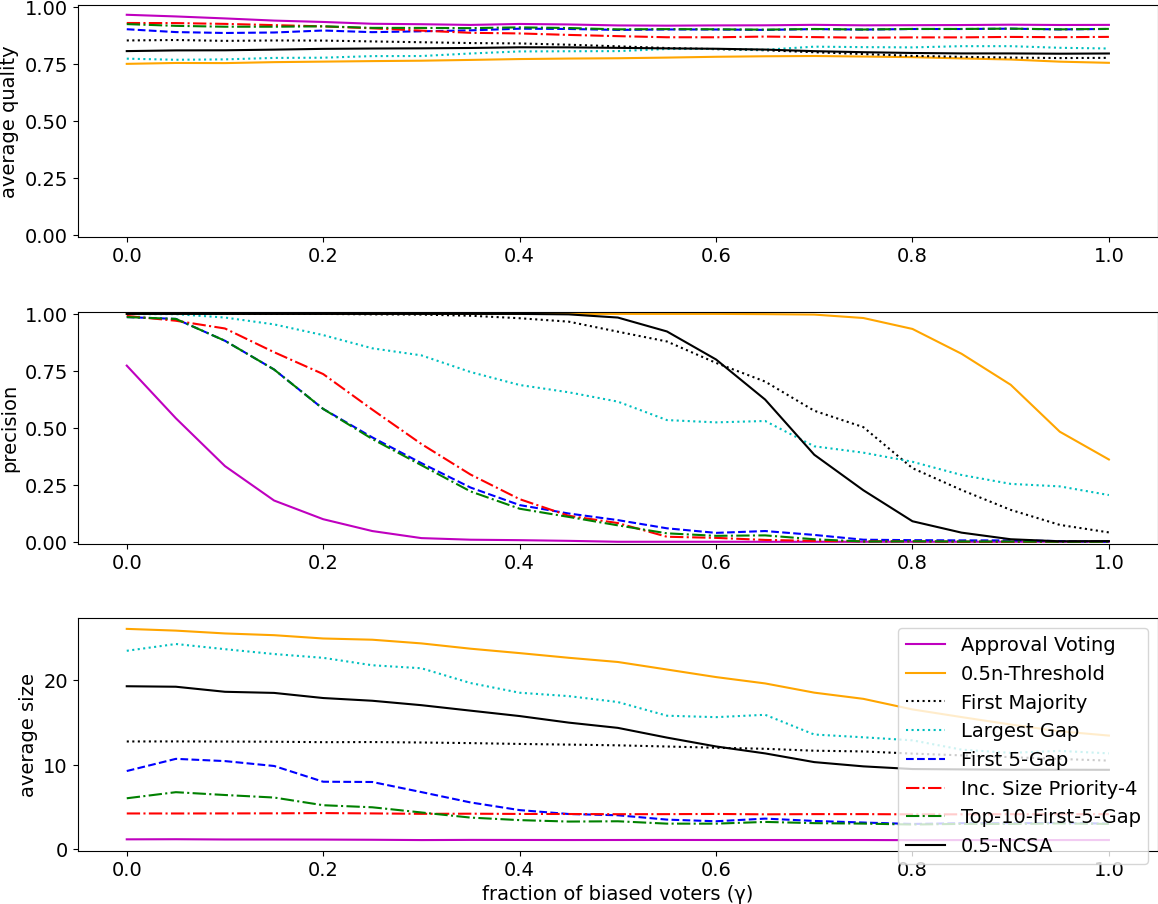

Supplement: Supplementary file 11 — (png 70 KB) [file 355_2023_1482_MOESM11_ESM.png]

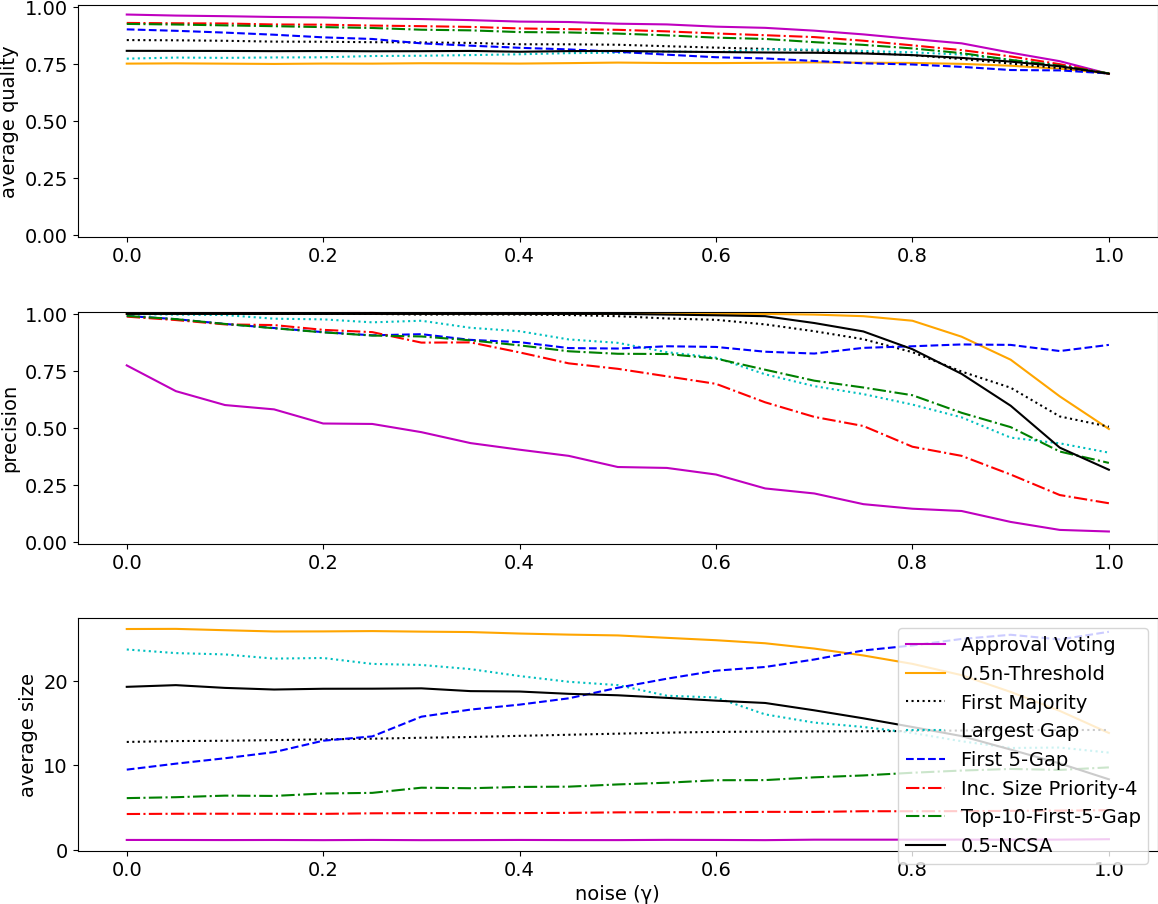

Supplement: Supplementary file 12 — (png 178 KB) [file 355_2023_1482_MOESM12_ESM.png]

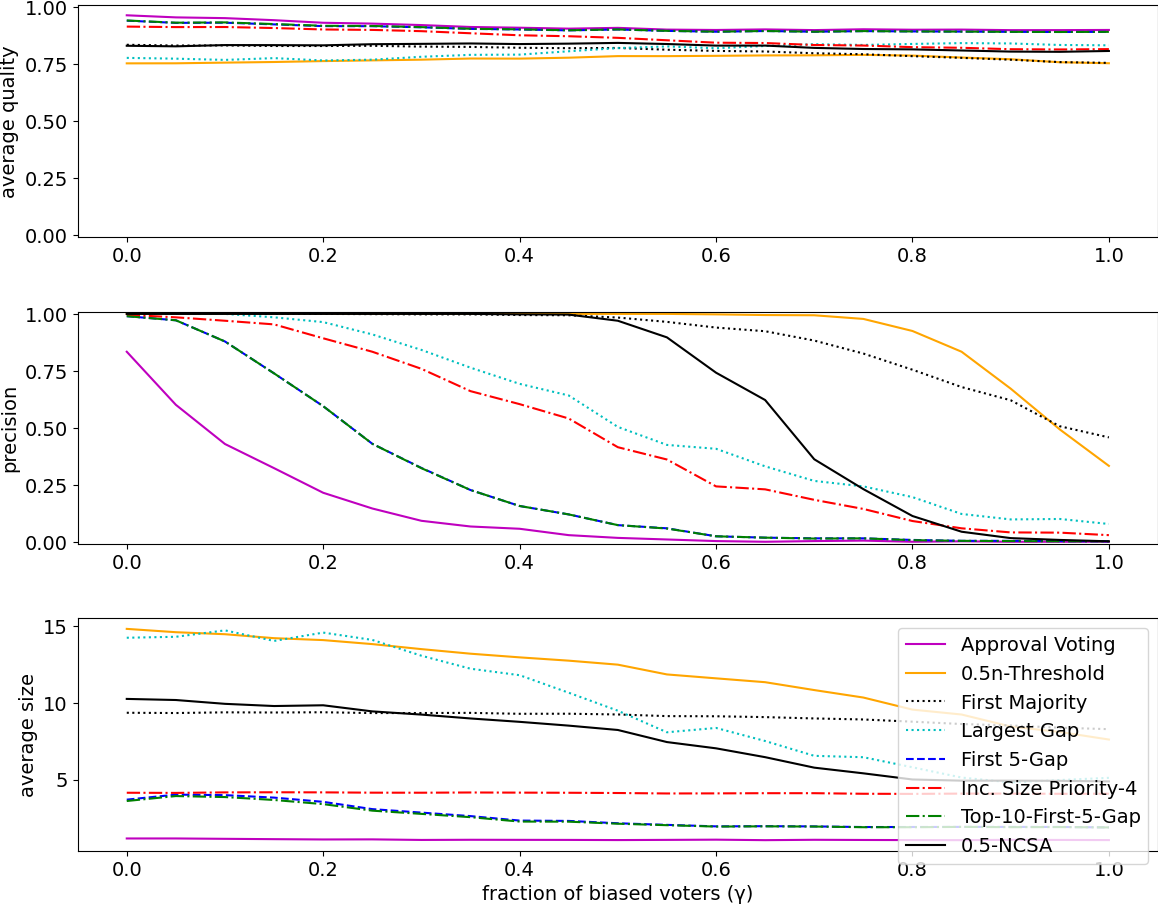

Supplement: Supplementary file 13 — (png 174 KB) [file 355_2023_1482_MOESM13_ESM.png]

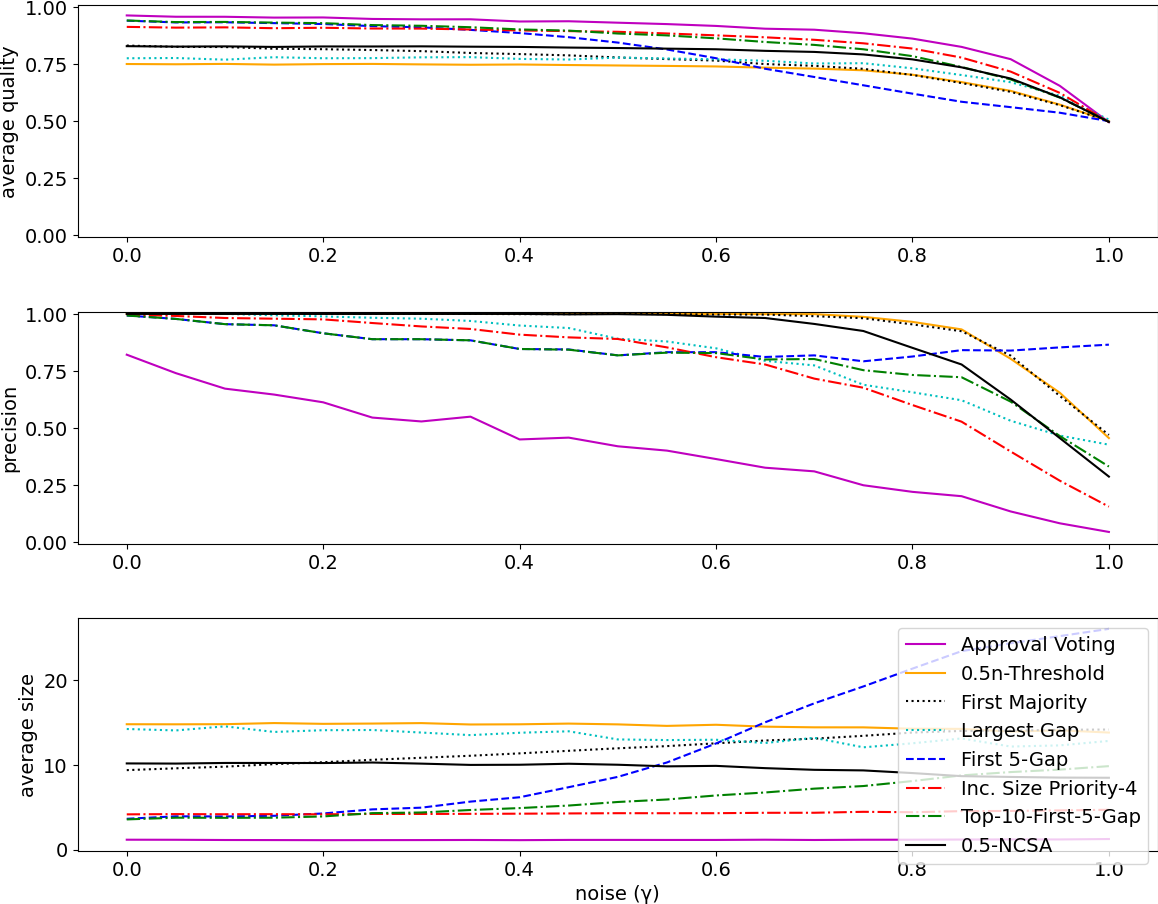

Supplement: Supplementary file 14 — (png 176 KB) [file 355_2023_1482_MOESM14_ESM.png]

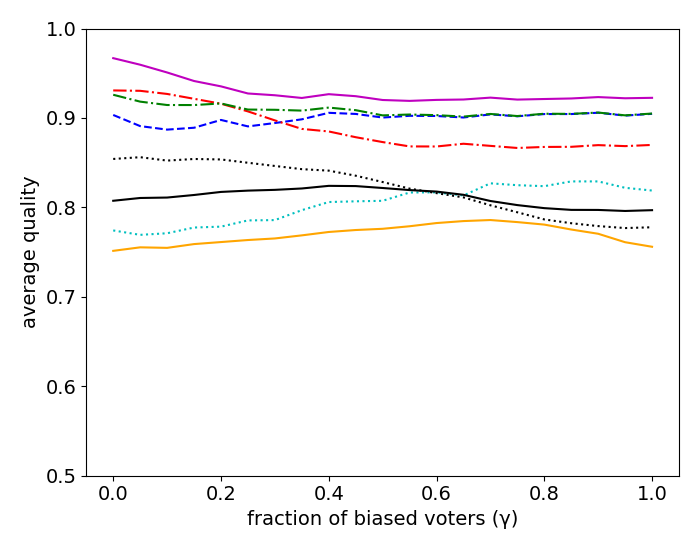

Supplement: Supplementary file 15 — (png 171 KB) [file 355_2023_1482_MOESM15_ESM.png]

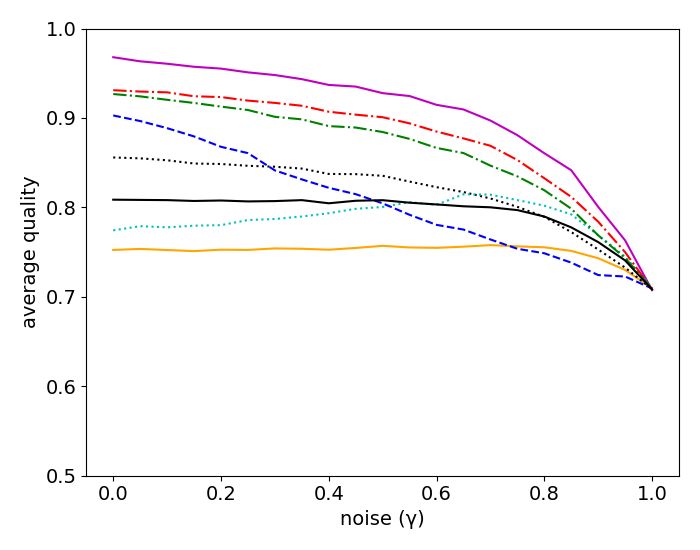

Supplement: Supplementary file 16 — (png 44 KB) [file 355_2023_1482_MOESM16_ESM.png]
